# On the Convergence and Capability of the Large-Eddy Simulation of Concentration Fluctuations in Passive Plumes for a Neutral Boundary Layer at Infinite Reynolds Number

**DOI:** 10.1007/s10546-020-00537-6

**Published:** 2020-06-26

**Authors:** Hamidreza Ardeshiri, Massimo Cassiani, Soon Young Park, Andreas Stohl, Ignacio Pisso, Anna Solvejg Dinger

**Affiliations:** 1 NILU-Norwegian Institute for Air Research, 2007 Kjeller, Norway; 2 Gwangju Institute of Science and Technology, Gwangju, 61005 South Korea; 3 Department of Meteorology and Geophysics, University of Vienna, 1010 Vienna, Austria

**Keywords:** Concentration moments, Plume dispersion, Large-eddy simulation grid resolution, Probability density function, Turbulence

## Abstract

Large-eddy simulation (LES) experiments have been performed using the Parallelized LES Model (PALM). A methodology for validating and understanding LES results for plume dispersion and concentration fluctuations in an atmospheric-like flow is presented. A wide range of grid resolutions is shown to be necessary for investigating the convergence of statistical characteristics of velocity and scalar fields. For the scalar, the statistical moments up to the fourth order and the shape of the concentration probability density function (p.d.f.) are examined. The mean concentration is influenced by grid resolution, with the highest resolution simulation showing a lower mean concentration, linked to larger turbulent structures. However, a clear tendency to convergence of the concentration variance is observed at the two higher resolutions. This behaviour is explained by showing that the mechanisms driving the mean and the variance are differently influenced by the grid resolution. The analysis of skewness and kurtosis allows also the obtaining of general results on plume concentration fluctuations. Irrespective of grid resolution, a family of Gamma p.d.f.s well represents the shape of the concentration p.d.f. but only beyond the peak of the concentration fluctuation intensity. In the early plume dispersion phases, the moments of the p.d.f. are in good agreement with those generated by a fluctuating plume model. To the best of our knowledge, our study demonstrates for the first time that, if resolution and averaging time are adequate, atmospheric LES provides a trustworthy representation of the high order moments of the concentration field, up to the fourth order, for a dispersing plume.

## Introduction

The dispersion of substances from a small punctiform source in an atmospheric turbulent flow is a physical phenomenon of capital importance in many ecological, environmental, and industrial applications. The concentration field of the dispersing substance displays a random behaviour that, for a specific point in space and time, can be fully characterized by its single-point probability density function (p.d.f.) (e.g., Pope 2000). The mean state of many processes with only a linear dependence on the concentration can be well characterized by the knowledge of the first moment of this p.d.f., the mean concentration. However in other cases, e.g., olfactory processes (Balkovsky and Shraiman 2002; Schauberger et al. 2011), or the release of toxic and flammable substances (Hilderman et al. 1999), non-linearity is observed. In such cases, knowledge of the higher moments of the concentration p.d.f. is needed. Certain modelling approaches such as the two-particle Lagrangian model (Durbin 1980; Thomson 1990; Franzese and Borgas 2002), the Eulerian model resolving the concentration-variance balance equation (Milliez and Carissimo 2008; Yee 2009), and heuristic Lagrangian methods based on single particle models (Ferrero et al. 2016; Cassiani 2013; Manor 2014) are able to estimate only the first two moments of the concentration. The meandering plume approach (Gifford 1959; Cassiani and Giostra 2002) can potentially estimate all concentration moments and the p.d.f.. However, the assumption behind the model formulation becomes less justified further away from the source where empirical parametrization and assumptions about the p.d.f. shape have to be made (Yee and Wilson 2000; Luhar et al. 2000; Cassiani and Giostra 2002; Franzese 2003; Mortarini et al. 2009; Marro et al. 2015). To the best of our knowledge, there are two modelling methods that potentially allow the explicit simulation of the concentration p.d.f. and its higher moments in real world conditions and at very high Reynolds numbers ($Re = U \delta /\nu$ with *U* a mean flow velocity scale, $\delta$ the boundary-layer height and $\nu$ the viscosity) typical of atmospheric flows. These models are the Lagrangian or Eulerian p.d.f. (micromixing) method (Pope 2000; Luhar and Sawford 2005; Cassiani et al. 2005a, b, c; Garmory et al. 2006; Cassiani et al. 2007, 2010; Postma et al. 2011; Amicarelli et al. 2012; Leuzzi et al. 2012) and the large-eddy simulation (LES) method (Henn and Sykes 1992; Sykes and Henn 1992; Weil et al. 2004; Xie et al. 2004a; Vinkovic et al. 2006; Xie et al. 2007; Philips et al. 2013).

Among these modelling approaches, LES is nowadays often viewed as the reference method to simulate the atmospheric flow and dispersion. Large-eddy simulation provides access to the three-dimensional turbulent flow field and it is sometimes used as a replacement for experimental measurements at high Reynolds number. Large scales of the turbulent flow are explicitly simulated by LES but the subfilter scales need to be parametrized using a subgrid scale (SGS) model (e.g., Deardorff 1973; Moeng 1984; Meneveau et al. 1996; Pope 2000; Celik et al. 2009). Implicit or explicit approaches to LES filtering are possible (e.g., Sagaut 2000; Celik et al. 2009) based on the decomposition of the unknown filtered correlations involving or not explicit filtering operations. In the context of implicit filtering the filter width enters into the modelling of the SGS stress tensor and depends on the grid size but it can still be made larger than the grid size to ensure results are independent of the grid and numerics (e.g., Mason and Callen 1986; Geurts and Frohlich 2002; Pope 2004; Geurts 2006). However, in most applications the filter is implicitly defined to be exactly or very close to the grid size, which makes the LES results dependent on the grid definition and on the numerical methods used (e.g., Pope 2004; Geurts 2006; Kemenov et al. 2012).

In engineering applications, various methods have been proposed to estimate the quality of the turbulent flow simulated by an LES model (e.g., Celik et al. 2005, 2009). However, we note that these methods are mostly suited for relatively low Reynolds number flows as they involve the actual viscosity of the fluid and convergence to direct numerical simulation (DNS) results. Large-eddy simulation of atmospheric flow is different as it involves the further assumption of infinite Reynolds number, totally neglecting the molecular viscous term in the equations, and the use of a wall-stress model (e.g., Moeng 1984; Porté-Agel et al. 2000; Chow et al. 2005; Sullivan and Patton 2011; Maronga et al. 2015). This implies that the viscous layer is totally unresolved or that the surface is rough but with unresolved roughness scale (Brasseur and Wei 2010).

The dispersion of scalars introduces further approximations associated with the numerical methods and SGS model used for the scalars. Subgrid-scale scalar fluctuations in LES can be obtained from algebraic and transport equation methods (e.g., Colucci et al. 1998; Pierce and Moin 1998; Jimenez et al. 2001; Balarac et al. 2008; Kaul et al. 2009). In atmospheric dispersion applications, the SGS scalar fluctuations are usually not explicitly modelled by a transport equation (e.g., Mironov et al. 2000; Heinze et al. 2015).

Despite the uncertainties related to the filter and numerical implementation, in recent years LES has become a tool used in many applied atmospheric dispersion studies, including the urban environment, and for critical applications such as the release of toxic gas substances (e.g., Fossum et al. 2012; Nakayama et al. 2013; Lateb et al. 2016). However, very few thorough evaluations of LES for plume dispersion and concentration fluctuations from small (point-like) sources in very high Reynolds number boundary layers are available. Henn and Sykes (1992) and Sykes and Henn (1992) studied dispersion and concentration fluctuations in the convective and neutral boundary layers, respectively. The maximum achievable resolution was limited by the available computing power, but source sizes smaller than grid resolution could be simulated by using a puff model for scalar dispersion with a semi-empirical parametrized SGS puff expansion (Sykes et al. 1984). This was empirically set by Sykes and Henn (1992) to match the Fackrell and Robins (1982) wind-tunnel experiment, thus allowing a satisfactory comparison with this dataset. Xie et al. (2004a, 2007) investigated plume dispersion and concentration fluctuations (including extremes) in a neutral boundary layer with the highest achievable LES grid resolution capable to resolve the scalar source size with one grid cell. More recently, Boppana et al. (2012) studied the relatively simpler case of dispersion from a line source in channel flow at a finite Reynolds number of 10800 and still underlines the difficulty implied in investigating scalar dispersion from small sources by means of LES, and the need of thorough validations.

Validating LES involving scalar fluctuations for a dispersing plume, at the very high Reynolds number typical of atmospheric flows, is a complicated task. For instance, the comparison with DNS is not feasible. Atmospheric dispersion experiments (e.g., Mylne and Mason 1991; Mylne 1992, 1993; Jørgensen and Mikkelsen 1993; Yee et al. 1993a, b; Mole and Jones 1994) providing concentration-fluctuation statistics have some characteristics that make them unsuitable for the purpose of validating LES results. These are, (1) the extremely small scalar sources used which are unreachable even for nowadays LES, (2) sensors located quite far downwind from the source so that the effects related to the source size have been mostly forgotten, and (3) emissions and measurements are located very close to the ground, where the effect of the wall-model parametrization in the LES are critical because most of the energy may not be explicitly resolved. A viable alternative, also used in previous LES studies that are mentioned above, is to use the few available wind-tunnel dispersion studies resembling atmospheric boundary-layer conditions (Fackrell and Robins 1982; Nironi et al. 2015). In these experiments the emitting sources are small compared to the boundary-layer thickness but within LES possibility (comparable to a few metres in the atmosphere) and are also placed at relatively high elevation where the LES explicitly resolves most of the turbulent kinetic energy (TKE). One general advantage of the wind-tunnel experiments compared to atmospheric measurements is that they are non-affected by unsteadiness and can use very long averaging time, which for higher moments of concentration is necessary. One potential disadvantage is that wind-tunnel data may be affected by an *Re* dependence if *Re* is not very high.

As recognized by many authors, evaluation of LES results should always involve a wide range of grid resolutions (e.g., Pope 2004; Celik et al. 2005; Klein 2005; Klein et al. 2008; Sullivan and Patton 2011; Kemenov et al. 2012) but these studies are rare in the literature. To our knowledge, there exists no study that systematically examines the grid dependence of the LES results for concentration fluctuations from a continuous small finite (point-like) source in an infinite-*Re* neutral boundary-layer configuration.

The tendency of the error to decrease towards zero by increasing grid resolution can potentially be investigated for low Reynolds number LES with explicit wall simulation that converge to a DNS solution when their resolution increases (e.g., Kemenov et al. 2012). However, as mentioned above, typical atmospheric LES are not part of this category because the Reynolds number is far above the reach of DNS feasibility. Nonetheless, exploring the dependence of the results on the grid resolution and comparison to experimental measurements are fundamental for determining whether the LES results for the scalar converge, in some sense, and to evaluating the expected range of variability in the results.

We take a heuristic but rational and comprehensive approach and explore many statistics, and characteristic scales of the velocity and scalar fields to find evidences of convergence in the turbulent fields of our LES. The analysis of the statistical characteristics of the fluctuating concentration field includes moments up to the fourth order and the concentration p.d.f. with an unprecedented level of detail for a LES. A clear indication on the range of validity of the Gamma p.d.f. model (e.g., Duplat and Villermaux 2008) for the concentration p.d.f. is also obtained. We use the freely available and widely used open source Parallelized LES Model (PALM) (Maronga et al. 2015) to make our analysis directly useful towards practical applications.

The paper is organized as follows. Section 2 provides a description of the numerical method and the simulated cases, and subsequently, turbulent flow statistics are discussed in detail in Sect. 3.1. The spectra of the TKE and two-point statistics are addressed in Sect. 3.2, and the analysis continues with the investigation of the scalar field. The mean concentration, concentration variance and its budget are discussed, respectively in Sects. 4.1 and 4.2, while the mechanisms generating concentration fluctuations, and how this are altered by not adequate resolution, are examined in Sect. 4.3. In Sect. 4.4, we examine the shape of the concentration p.d.f. using the scaled moments of scalar concentration, including the ratio of standard deviation and mean, the skewness and the kurtosis. Finally, a summary and discussion are presented in Sect. 5.

## Methods

PALM is an open source model code developed mainly at University of Hannover (Maronga et al. 2015). Here the code is used to solve the non-hydrostatic, filtered incompressible Navier–Stokes equations in Boussinesq-approximated form at formally infinite Reynolds number ($Re = u_\infty \delta /\nu$, with $u_\infty$ the freestream velocity) due to the neglect of molecular viscous stress (e.g., Geurts and Frohlich 2002; Stevens et al. 2014), with an advection–diffusion equation for the transport of a passive scalar. Details of the governing equations are reported in Appendix 1 for completeness.

For the advective term in the incompressible LES model equations, the Piacsek and Williams (1970) second-order, formally energy conserving scheme, was chosen over the fifth-order dissipative scheme proposed by Wicker and Skamarock (2002). Heinze et al. (2015) compared these two schemes in PALM and found that the Wicker and Skamarock (2002) scheme is much more dissipative, artificially reducing the scale-interaction term and therefore the transfer of kinetic energy from the resolved scale part of the energy spectrum to the subgrid scale part. Indeed the use of high-order finite-difference dissipative schemes in LES generates excessive damping of the high frequencies and often shows numerical dissipation larger than subgrid scale dissipation (e.g., Beudan and Moin 1994; Mittal and Moin 1997; Sagaut 2000; Park et al. 2004). This can be avoided if the mixing length is made suitably larger than the grid size (e.g., Mason and Callen 1986) but this is seldom done, especially in practical applications, as grid resolution is usually at the limit of available computational resources. On the other hand the Piacsek and Williams (1970) second-order scheme has been successfully used in LES (e.g., Sykes and Henn 1988; Cuijpers and Duynkerke 1993; Mason and Brown 1999; Brown et al. 2000; Dosio et al. 2003). For the dispersing scalar plume, PALM allows the use of two possible schemes. The monotone, locally modified version of Bott’s advection scheme proposed by Chlond (1994) is used, ensuring positive definite scalar values and mass conservation. The non-monotone scheme of Wicker and Skamarock (2002) is also available, but was found to be inadequate for simulating plume dispersion

A three-dimensional domain of $4.8~\text {m} \times 0.8~\text {m} \times 0.8~\text {m}$, respectively in the along-wind (*x*), crosswind (*y*) and vertical (*z*) directions is simulated to reproduce the wind-tunnel experiment of Nironi et al. (2015) where a boundary-layer thickness of $\delta = 0.8~\text {m}$ was measured. Some aspects of the turbulent field are also comparable to the wind-tunnel experiment of Fackrell and Robins (1982) (hereafter F&R) if appropriately scaled, as extensively discussed in Nironi et al. (2015). Therefore, the measurements of F&R will also be used when possible.

The neutral boundary layer is simulated as an incompressible half-channel flow at infinite Reynolds number with a strictly symmetric, stress-free condition at the top of the domain ($\partial u/\partial z = 0, \partial v/\partial z = 0$). The flow is driven by a constant mean pressure gradient. Although this flow configuration is not realizable in nature, it is often used in order to simulate wind-tunnel-generated boundary layers and the atmospheric boundary layer, neglecting Coriolis effects (e.g., Schumman 1989; Porté-Agel et al. 2000; Xie et al. 2004b; Cassiani et al. 2008; Brasseur and Wei 2010; Margairaz et al. 2018). The constant mean pressure gradient is defined as $\partial p / \partial x = -u_{*}^{2} / \delta$, where $u_* = 0.185~{\text {m s}}^{-1}$ is the friction velocity measured in the wind tunnel. On the bottom wall the roughness length $z_0 = 1.1 \times 10^{-4}~\text {m}$ is also set equal to that estimated in the wind tunnel of Nironi et al. (2015). The roughness length enters in the wall model as the wall is not explicitly resolved but a constant-flux layer is used as is commonly done in atmospheric simulations (e.g., Moeng 1984; Maronga et al. 2015). For the velocity, periodic boundary conditions are used on the lateral boundaries, while non-periodic boundary conditions are set for the passive scalar (see Appendix 2 for more information about the boundary conditions).

The number of nodes of the computational grid ($N_x \times N_y \times N_z$, with $N_x$, $N_y$, and $N_z$ being the number of grid points in along-wind, crosswind, and vertical directions, respectively) is ranging from $256\times 64 \times 64 \approx 10^6$ nodes to $2048 \times 512 \times 512 \approx 5 \times 10^8$ nodes. The size of the source is $d_s=12~\text {mm}= 0.015 \delta$ in the vertical and crosswind directions. This initial source size is in the middle of the range of the source sizes investigated in F&R, where $d_s / \delta = 0.0025, 0.007, 0.0125, 0.0208, 0.0291$ were considered, but it is larger than the range investigated in Nironi et al. (2015), $d_s / \delta =0.00375$ and 0.0075. This initial source size was chosen so that the grid convergence of the statistics is fully investigated among four different grid resolutions used to resolve the flow. The emission source is simulated with exactly one grid cell in $N_x=256$, $2^3$, $4^3$, and $8^3$ grid cells in $N_x=512$, $N_x=1024$, and $N_x=2048$ simulations, respectively. Hereafter, the four simulations are referred to according to their $N_x$ value. The source is located at $y_s$, corresponding to the centre of computational domain in crosswind direction, and at the elevation of $z_s=0.19\delta$ which corresponds to the elevation used in both Nironi et al. (2015) and F&R. Table 1 lists some important quantities of the wind-tunnel and numerical experiments.

For a horizontally homogeneous boundary-layer flow discussed here, both time averages and plane averages can be used to calculate one-point flow statistics. However, the scalar field from a small (point-like) source is fully non-homogeneous with only a statistical symmetry, with respect to source location along the *y* direction, potentially usable to increase the samples. In the following, we will use only time averages to calculate the scalar statistics unless otherwise explicitly stated and explained. Large averaging times are necessary to obtain convergence in higher-order statistical moments if the plane average is not used. The averaging time used for the statistics is 150 s, i.e. between 600 and 800 times the estimated Lagrangian time scale as further discussed below. A spin-up time of 120 s was used to ensure that flow statistics are in steady state before starting the time average. These spin-up and averaging times imply that about $8 \times 10^5$ core hours were used typically for one 2048 simulation.

In the following we adopt a standard notation with the overbar $\bar{()}$ denoting a resolved scale (filtered) variable, the single prime $()^{\prime }$ a subfilter scale fluctuation, the angle brackets $\left\langle () \right\rangle$ a space and/or time average and the double prime ${()}^{^{\prime \prime }}$ a fluctuation from this average. Any flow variable $\phi$ can be decomposed as: $\phi = \left\langle {\bar{\phi }} \right\rangle + {{\bar{\phi }}}^{^{\prime \prime }} + \phi ^{\prime }$. Meteorological or index notation are used as convenient so $u_1=u, u_2=v, u_3=w$ represent the velocity components in the along-wind $x_1=x$, crosswind $x_2=y$, and vertical $x_3=z$, directions respectively. Vectors are represented in bold characters, e.g. $\mathbf{x }=(x_1,x_2,x_3)$.

## Turbulent Velocity Field

To correctly interpret the scalar dispersion it is necessary to understand the statistical characteristics of the velocity field. Here the first- and second-order statistical moments of velocity and turbulent structures are examined, including their change with grid resolution.

**Table 1:** Boundary-layer characteristics: freestream velocity $u_\infty$, friction velocity $u_*$, boundary-layer thickness $\delta$, roughness length $z_0$, mean velocity at source elevation $u_s$, Reynolds number of the flow $Re = u_\infty \delta /\nu$, Reynolds number based on SGS viscosity ($\nu _{SGS}$) in the middle of the boundary layer, $Re_{\nu _{SGS}} = u_*\delta /\nu _{SGS}$

Study	$u_\infty \,({\text {m s}}^{-1})$	$u_*\,({\text {m s}}^{-1})$	$\delta \,(\text {m})$	$z_0\,(\times 10^{-4}\text {m})$	$u_s\,({\text {m s}}^{-1})$	*Re*	$Re_{\nu _{SGS}}$
Nironi	5	0.185	0.8	1.1	$\sim 3.33$	260,000	
F & R	4	0.188	1.2	2.9	$\sim 3$	310,000	
$256 \times 64 \times 64$	4.83	0.185	0.806	1.1	3.65	$\infty$	1500
$512 \times 128 \times 128$	4.88	0.184	0.803	1.1	3.70	$\infty$	3900
$1024 \times 256 \times 256$	4.81	0.184	0.802	1.1	3.78	$\infty$	10,400
$2048 \times 512 \times 512$	4.86	0.184	0.800	1.1	3.87	$\infty$	28,000

**Fig. 1:**
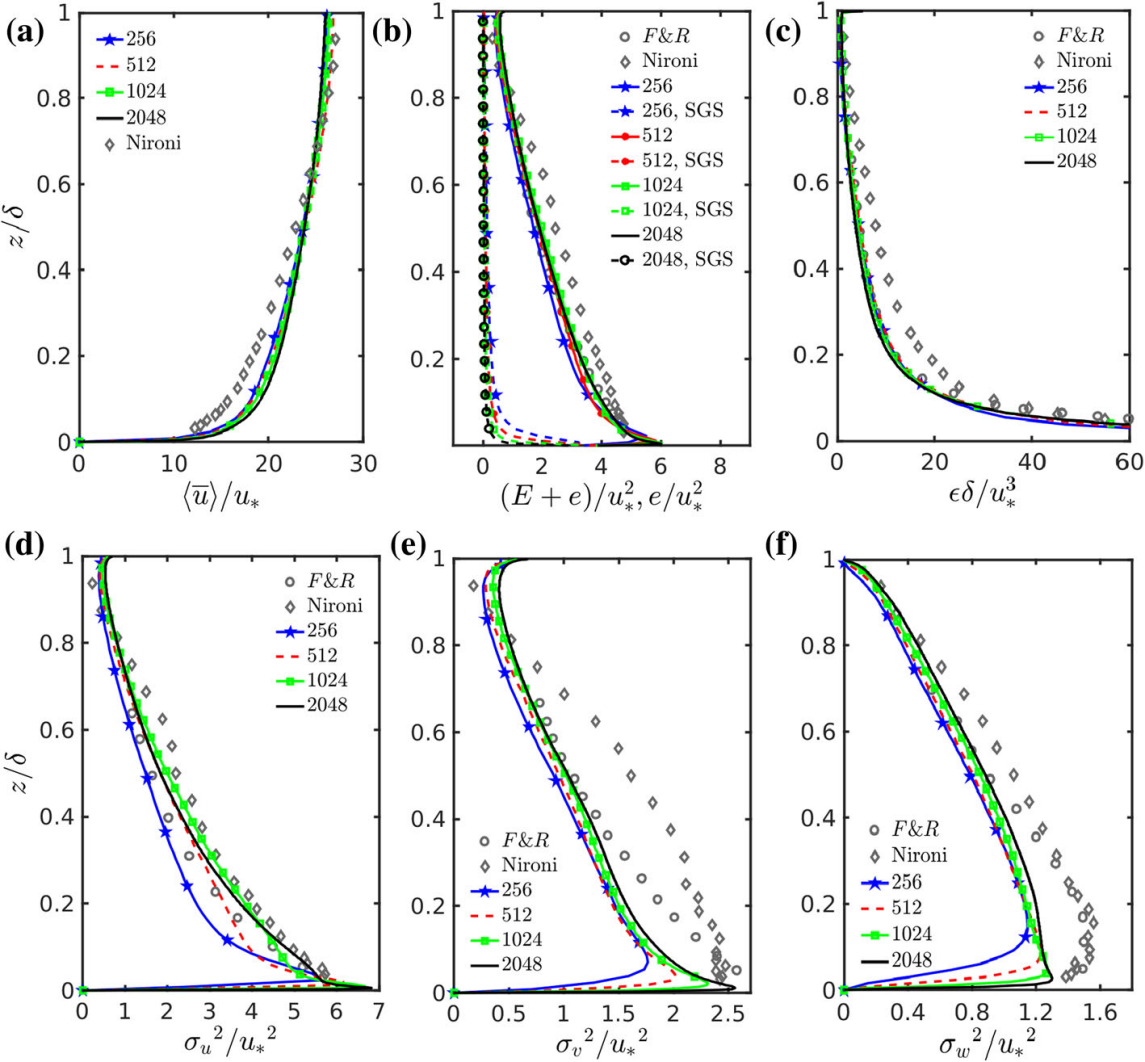
**a** Mean wind velocity profile scaled by friction velocity. **b** Total and SGS TKE as a function of height. **c** Dissipation rate from the residual of the resolved scale TKE balance (Eq. 1). Normalized variances of the resolved scale **d** along-wind, **e** crosswind and **f** vertical velocity components

### Turbulent Flow Statistics

The LES flow is compared with the wind-tunnel measurements of Nironi et al. (2015) and F&R. Averaging is done over the horizontal plane and in time. The mean wind speed at the top of the boundary layer ($u_\infty$) is equal to $4.8~{\text {m s}}^{-1}$ in the LES; it is $5~{\text {m s}}^{-1}$ in Nironi et al. (2015), and $4~{\text {m s}}^{-1}$ in F&R, as listed in Table 1. We remark that the Nironi et al. (2015) experiment has the same values for roughness length, boundary-layer thickness and a very similar friction velocity as in our simulations and should also have very similar mean wind profiles having very similar $u_\infty$.

Figure 1a shows the mean wind profile $\left\langle {\bar{u}} \right\rangle$ scaled by friction velocity. The LES results are weakly sensitive to resolution with small but visible changes. By increasing the grid resolution, the mean wind speed increases at the source elevation, although it tends to be similar at the top of the boundary layer (see Table 1). The LES overestimates mean wind speed profiles compared to the wind-tunnel measurements in the lower part of the domain. This overestimation of mean wind speed in LES using wall models, compared to the logarithmic law profile, has been investigated by many authors (Xie et al. 2004b; Brasseur and Wei 2010; Hultmark et al. 2013; Ercolani et al. 2017) and some correction methods have been proposed (e.g., Xie et al. 2004b; Hultmark et al. 2013). The factors influencing this aspect are the SGS model, the grid aspect ratio and the wall model. Based on the SGS model closure, grid resolution and grid aspect ratio, all the simulations here but the coarsest fall in the high accuracy zone estimated by Brasseur and Wei (2010), their Fig. 7, in relation to the LES capability of predicting the logarithmic law of the wall. More recent literature (Ercolani et al. 2017) found that the SGS model used here gives overestimation of mean wind speed when used with an almost isotropic grid aspect ratio between one and two, and this is confirmed by our results. Ercolani et al. (2017) suggested that the overestimation is linked to the over dissipative nature of the SGS model and that this behaviour is somewhat masked by the use of an anisotropic grid with an aspect ratio of $(\Updelta x= \Updelta y) / \Updelta z=4$. This was not attempted here as the recommended aspect ratio is not ideal for the plume dispersion modelling that ideally requires the crosswind and vertical grid size to be the same. The wall model used in PALM (see Appendix 2) assumes that the log law is valid for the instantaneous resolved wind velocity magnitude. Wall models belonging to this class are commonly used in atmospheric LES (e.g., Moeng 1984; Maronga et al. 2015) and are known to overestimate the mean wind speed compared to the log law as discussed in Hultmark et al. (2013). However, again from the plume dispersion perspective, which is our main focus, the difference in the mean wind speed at source elevations is small and this is the main advection velocity for plumes.

The difference of mean wind speed among different resolutions is quite small here, but we mention that an aspect that influences the convergence (among grid resolutions) of mean wind profiles in the wall-model LES is the height used to inject information in the wall model (Kawai and Larsson 2012). The PALM code uses the standard LES approach and takes advantage of the available grid resolution feeding information to the wall model at $\Updelta z/2$. Kawai and Larsson (2012) found that convergence is improved if the information is transferred to the wall model at a constant height irrespective of grid resolution.

Figure 1b shows the SGS and total TKE. The SGS TKE ($e = \frac{1}{2} \overline{u_i'u_i'}$) is calculated in PALM by solving a model equation (e.g., Moeng 1984; Maronga et al. 2015) as it is common in many atmospheric LES models. The total TKE is $E+e$ where *E* is the resolved scale TKE, $E = \frac{1}{2} \left\langle {{\bar{u}}_i}^{{\prime \prime }} {{\bar{u}}_i}^{{\prime \prime }} \right\rangle$. The total TKE is in very good agreement with both experimental datasets at all grid resolutions. Pope (2000) suggested that LES is properly resolved if at least $80\%$ of TKE is resolved, and for LES with wall modelling this is possible only away from the surface. For the current simulations this condition is fulfilled for $z / {\delta } > rapprox 0.05, 0.02, 0.01, 0.005$, respectively, moving from coarser to finer resolved simulations. Therefore, at the elevation where the plume is released most of the energy is explicitly resolved in all cases. This also means that numerical dissipation plays an important role.

Figure 1c shows the mean dissipation rate ($\epsilon _{E}$) of TKE as obtained from the residual of the resolved TKE balance, Eq. 1,(e.g., Mironov et al. 2000),

1 $$\begin{aligned} \frac{\partial E}{\partial t} = 0 = -\left\langle {{\overline{u}}'' {\overline{w}}''} \right\rangle \frac{\partial \left\langle {\overline{u}}\right\rangle }{\partial z} - \frac{\partial \left\langle {\overline{w}}''E \right\rangle }{\partial z} - \frac{1}{\rho } \frac{ \partial \left\langle {\overline{w}}'' {\overline{p}}'' \right\rangle }{\partial z} - \frac{2}{3} \frac{ \partial \left\langle {\overline{w}}'' e'' \right\rangle }{\partial z} - \epsilon _{E}, \end{aligned}$$

where horizontal homogeneity and steady state have been used. In this equation, *p* represents the perturbation pressure (see e.g. Moeng 1984; Maronga et al. 2015, and Appendix 1 for more details). The dissipation rate $\epsilon _{E}$, is the last term on the right-hand side (r.h.s), and has been evaluated as a residual. The agreement between this estimation of the dissipation and the experimental value of F&R is extremely good. The experimental values of Nironi et al. (2015) shows a just slightly higher dissipation. We remark again that the values in Fig. 1c pertain to elevations where most of the TKE is resolved ($z / \delta \ge 0.05$) and therefore Eq. 1 is well representative of the total TKE. We found that other estimates of the mean dissipation are not representative of the actual dissipation. This agrees with Heinze et al. (2015), who used the PALM code to compare three methods of estimation of the TKE dissipation; (i) the parametrized form in the SGS TKE equation

2 $$\begin{aligned} {\epsilon }_{SGS} = \left( 0.19 + 0.74 \frac{l}{\Updelta } \right) \frac{e^{\frac{3}{2}}}{l}, \end{aligned}$$

where $\Updelta =\root 3 \of {\Updelta x \Updelta y \Updelta z}$ with $\Updelta x, \Updelta y, \Updelta z$ being the grid spacing in the *x*, *y* and *z* directions, respectively and *l* is the SGS mixing length; (ii) the computation of the scale-interaction term describing the transfer of energy between resolved and subgrid scales (Brown 1994; Heinze et al. 2015; Mironov and Sullivan 2016); and (iii) the residual of the TKE budget. They found that the only reliable method to compute energy dissipation is from the residual of the TKE budget. This is not surprising, as the latter method includes numerical dissipation, which can be a dominating fraction of dissipation where LES resolves most of the TKE. This also agrees with Glendening and Haack (2001) who compared several numerical schemes and found that in the presence of strong mean advection only pseudo-spectral methods were able to correctly avoid spurious dissipation at high wavenumbers. We note that Heinze et al. (2015) used the total TKE balance, but for the elevation considered here most of the energy is resolved. For values $z / {\delta } \lesssim 0.05, 0.02, 0.01, 0.005$, respectively, moving from coarser to finer resolved simulations the SGS dissipation starts to dominate over the resolved scales (not shown here).

Figure 1d–f show the scaled resolved variance of the three velocity components. The variance of the resolved scale along-wind component, $\sigma ^2_{u}$, progressively gets closer to the experimental values when increasing the grid resolution, and for the highest resolutions the agreement is very good. The crosswind and vertical velocity variance similarly become closer to the wind-tunnel experimental values when increasing the grid resolution, but the agreement is not as good as for the along-wind velocity component. Similar values of crosswind velocity variance were obtained in the LES of Xie et al. (2004a) using the SGS mixed-scale model of Sagaut (1995). Porté-Agel et al. (2000) obtained similar values of crosswind velocity component using a Smagorinsky model closure, but higher values using a scale dependent dynamic model. The two wind-tunnel experiments have very similar values, except for the crosswind component (*v*), where F&R is more similar to the LES results compared to Nironi et al. (2015). The Reynolds stress, $\langle {\bar{u}}^{\prime \prime } {\bar{w}}^{\prime \prime } \rangle$ (not shown here) is a straight line as expected and matches very well the experimental data. In the highest resolution case, the observations are nearly captured, even without the contribution of the SGS stress. Obviously in the lowermost layer close to the wall everything is subgrid.

It can be anticipated that by considering the difference in the crosswind and vertical velocity components variance (Fig. 1e, f), a lower plume spread may be expected for the LES when compared to the wind-tunnel data of Nironi et al. (2015). However, overall the second moments of the high resolution LES are similar to the wind-tunnel experimental values. Plume dispersion statistics in neutral boundary layers are mainly driven by second-order velocity statistics and by velocity length and time scales, which are the footprint of turbulent velocity structures. In the next section, we complete the velocity field analysis using the spectrum, two-point correlation and turbulent length scales across LES resolutions before turning our attention to the scalar field.

### Turbulent Flow Structures and Length Scales

**Fig. 2:**
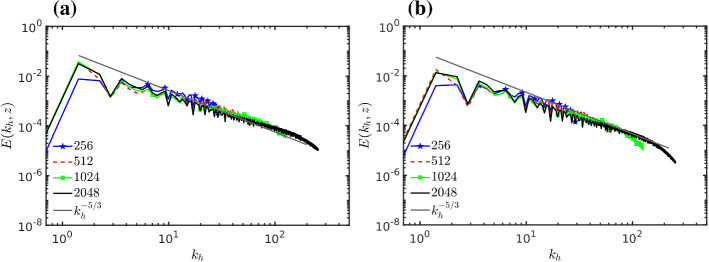
Two-dimensional spectrum of TKE $E(k_h,z)$ for different grid resolutions with the $k_h^{-5/3}$ scaling at, **a** source elevation, and **b** in the middle of the boundary layer

To examine the existence and extension of the inertial subrange, the two dimensional spectra of the TKE on the horizontal plane are now considered. As discussed in Sullivan and Patton (2011) the two-dimensional spectra are more representative of the spatial eddy scale than one-dimensional spectra. Figure 2 shows the time-averaged spectrum of the TKE $E(k_h,z)$ (e.g., Pope 2000), where $k_h$ is here the horizontal dimensionless wave vector module $k_h = k \delta (2\pi )^{-1}$with *k* being the horizontal angular wave vector module (${\text {rad m}}^{-1}$) (e.g., Wyngaard 2010). The spectrum is obtained by integrating circles of increasing radius and it is presented for all grid resolutions both at the source elevation, $z=0.19 \delta$, and at the middle of the boundary layer, $z=0.5 \delta$. The $k_h^{-5/3}$ Kolmogorov inertial subrange scaling is also shown as a straight line. The inertial subrange is visible only in the two higher resolved simulations (1024 and 2048), which therefore can be assumed to converge in this sense. The lowest resolution seems especially under-resolved, lacking the display of an inertial subrange.

Large-scale turbulent structures are fundamental for plume dispersion as they define the persistence of spatial and time correlations. In an inhomogeneous and anisotropic shear flow a variety of integral length scales exists to describe these structures. These length scales can be estimated from the two-point spatial correlation coefficient, which is defined as (e.g., Carlotti and Drobinski 2004; Nironi et al. 2015)

3 $$\begin{aligned} \rho _{ij}(\mathbf{x}, r ) = \frac{\left\langle {\overline{u}}_i''(\mathbf{x }) {\overline{u}}_j ''(\mathbf{x} + r ) \right\rangle }{ \left\langle {\overline{u}}_i'' {\overline{u}}_j'' \right\rangle }, \end{aligned}$$

where $\mathbf{x }$ is a fixed point and $\mathbf{r }$ is a generic vector. In vertically inhomogeneous turbulence, the two-point correlation depends on the vertical position. However, the most significant position to characterize plume dispersion for an elevated small (point-like) source is certainly that of the source. Therefore, the analysis here is about the correlations centred at source position and limited to the single velocity component correlation coefficients, $\rho _{ii}$ (no summation implied in repeated index here), which are used to obtain the relevant integral length scales. As extensively discussed in Carlotti and Drobinski (2004) many integral scales can be defined in inhomogeneous anisotropic turbulence by integrating the spatial correlation coefficient over the distance $\mathbf{r }$ along all the possible orthogonal directions,

4 $$\begin{aligned} L^{(+/-)}_{ii,j}(\mathbf{x }_s) = \int _{0}^{\infty } \rho _{ii}(\mathbf{x }_s, \pm r \mathbf{e }_j)dr = \int _{0}^{\infty } \frac{\left\langle {\overline{u}}_i''(\mathbf{x }_s) {\overline{u}}_i ''(\mathbf{x }_s \pm r \mathbf{e }_j ) \right\rangle }{ \left\langle {\overline{u}}_i'' {\overline{u}}_i'' \right\rangle } dr, \end{aligned}$$

where $\mathbf{e }_j$ is the unit vector in *x*, *y*, *z* directions; $\mathbf{x }_s$ is the source position; and no summation is implied in repeated index. In the present case of wall bounded flow, the only non homogeneity and possible asymmetry is in the vertical direction (Carlotti and Drobinski 2004). Following Nironi et al. (2015) our analysis is limited to $L_{uu,x}=L_{11,1}$, $L_{vv,y}=L_{22,2}$ and $L_{ww,z}=L_{33,3}$, with the latter two being the more relevant for understanding plume dispersion in the crosswind and vertical directions (Nironi et al. 2015). The computed length scale values are reported in Table 2. The values of the length scales are obtained by fitting the exponential function of the form of $\exp (\mp r/L_{ii,j})$ to the profiles of the correlation coefficient, where the signs depend on the direction being positive or negative, respectively. These profiles must be symmetric except for the vertical correlation coefficient ($\rho _{33}$) where the wall effect causes asymmetry. Considering the average value of the length scales in along-wind and crosswind directions, the Table shows that the 2048 simulation has more persistent correlations with longer length scales in all directions compared to the 512 and 1024 simulations. The low resolution (256) simulation shows different characteristics, having a much smaller $L_{uu,x}$ and being the only one with $L_{ww,z}> L_{vv,y}$ and having larger $L_{ww,z}$ compared to the 2048 simulation. This further shows that this simulation is under resolved.

**Table 2:** Estimate of the Eulerian length scales from the two-point spatial correlation coefficient, by fitting the exponential function of the form $\exp (\mp r/L_{ii,j})\mathbf{e }_j$ to the profiles of the correlation coefficient. The first value in $L_{ww,z}$ column corresponds to the positive distance $+r\mathbf{e }_j$ and the second value is for negative distance $-r\mathbf{e }_j$ as specified in Eq. 4

Resolution	$L_{uu,x}(\text {m})$	$L_{vv,y}(\text {m})$	$L_{ww,z}^{(+/-)}(\text {m})$
$256 \times 64 \times 64$	0.239	0.080	0.089–0.064
$512 \times 128 \times 128$	0.669	0.068	0.067–0.049
$1024 \times 256 \times 256$	0.740	0.068	0.063–0.046
$2048 \times 512 \times 512$	0.919	0.098	0.076–0.048

**Fig. 3:**
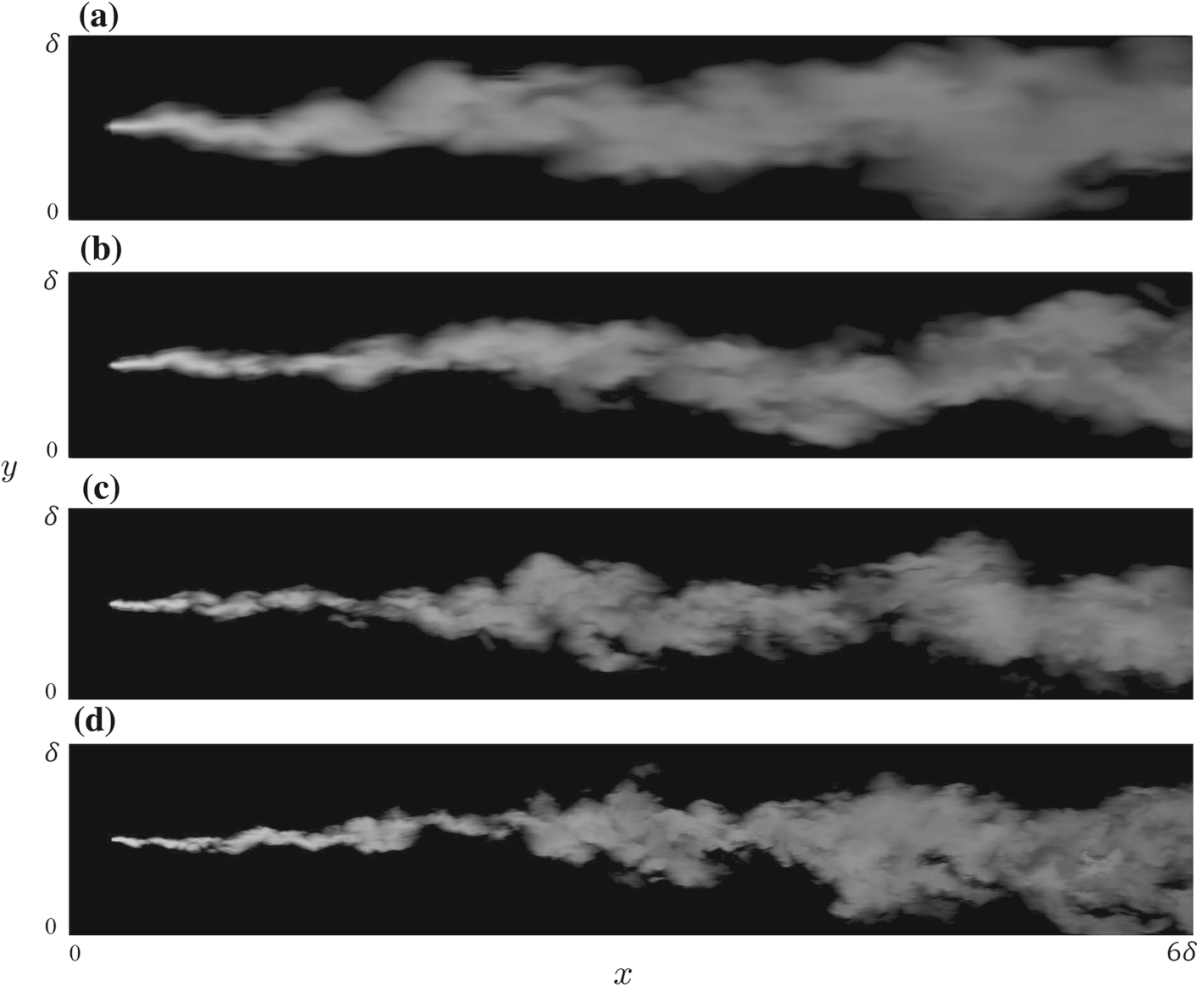
Snapshots of the turbulent scalar field in (*x*, *y*) plane at the source elevation for **a** 256, **b** 512, **c** 1024, and **d** 2048 simulation. The plot shows a range of $0<x<6\delta$ and $0<y<\delta$. Note that in the following, $x=0$ corresponds to the emission point

**Fig. 4:**
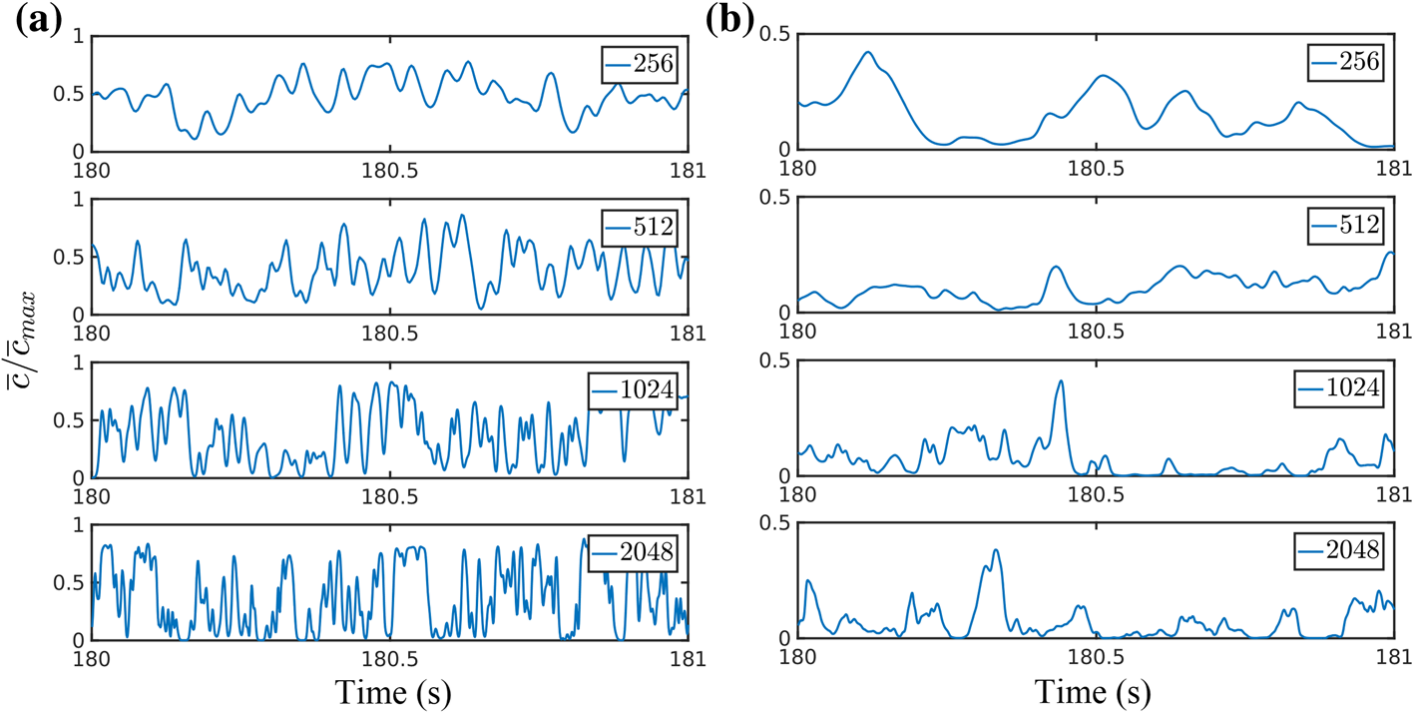
Time series of the normalized scalar concentration for different grid resolutions at, **a**$x/\delta = 0.13$, and **b**$x/\delta = 3.75$, for a period of 1 s. Here ${\bar{c}}_{max}$ is the maximum concentration of the time series data (over an averaging period) of the corresponding resolutions

## Turbulent Scalar Field

The transport of the passive scalar in PALM is simply obtained by solving an advection diffusion equation for the resolved scalar field,

5 $$\begin{aligned} \frac{\partial {\overline{c}}}{\partial t} = -\frac{\partial ({\overline{u}}_i {\overline{c}})}{\partial x_i} + \frac{\partial }{\partial x_i}\left( K_s \frac{\partial {\overline{c}}}{\partial x_i} \right) , \end{aligned}$$

where the SGS scalar diffusivity is defined as $K_s=\left( 1+\frac{2l}{\Updelta } \right) K_m$, and $l=\text {min}(1.8z,\Updelta )$ (Moeng and Wyngaard 1988; Maronga et al. 2015). As remarked in Sect. 2, the Bott advection scheme as modified by Chlond (1994) has been used in this study. Figure 3 shows snapshots of the concentration field for all the resolutions on the (*x*, *y*) plane at $z=z_s=0.19\delta$. There are obvious visual differences in the scalar field up to 1024 resolution, while between 1024 and 2048 these differences become more subtle. Interestingly, this visual impression is somewhat confirmed by the quantitative analysis below. Time series of the concentration at the location of maximum mean concentration are shown in Fig. 4 for $x/\delta =0.13$ and $x/\delta =3.75$. Similarly to what was observed in the snapshots, the differences are rather obvious up to 1024 resolution and become more subtle between 1024 and 2048.

### Mean Concentration Field

**Fig. 5:**
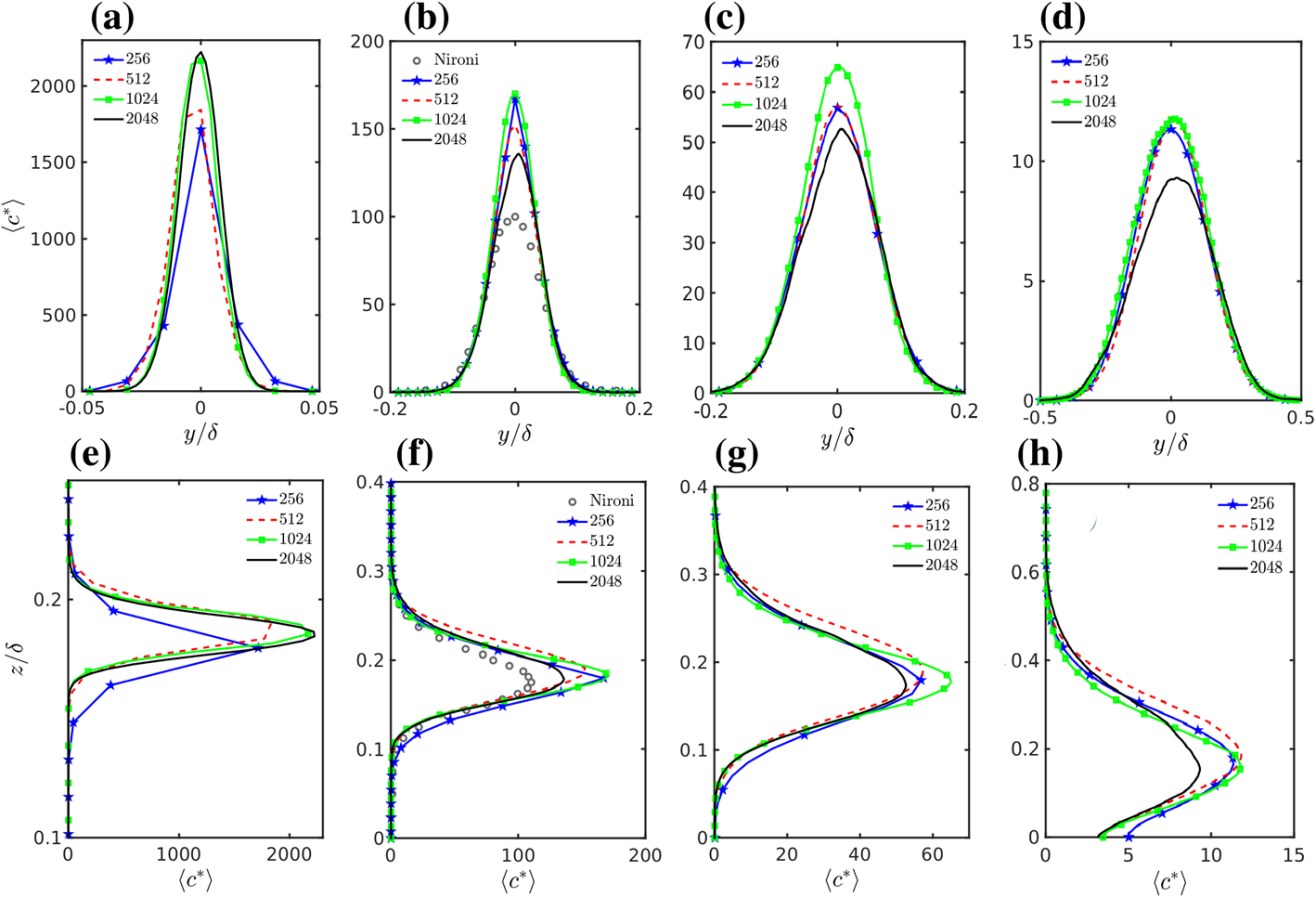
**a**–**d** Transversal, and **e**–**h** vertical profiles of mean concentration at various downwind distances, **a**, **e**$x^*/\delta =0.13$, **b**, **f**$x^*/\delta =0.725$, **c**, **g**$x^*/\delta =1.25$ and **d**, **h**$x^*/\delta =3.75$. Experimental data are extracted from Nironi et al. (2015)

Crosswind and vertical profiles of the mean concentration $\left\langle {\overline{c}} \right\rangle$, through its maximum for different grid resolutions at various downwind distances are plotted in Fig. 5. Angle brackets represent only the time average when the scalar is involved. The concentrations are reported following Nironi et al. (2015) by using the scaling ${\overline{c}}^{*} = {\overline{c}} (u_s \delta ^2 / Q)$ where $Q = 1~{\text {kg s}}^{-1}$ is the mass flow rate at the source located at $z_s$. We would expect that the mean concentration is minimally sensitive to the grid resolution in LES, as the mean concentration is influenced by the whole turbulent spectrum and the LES should explicitly capture most of the energy. Contrary to this intuitive picture some difference among grid resolutions is visible. The 2048 simulation stands out with a much higher spread and lower peak concentrations compared with lower grid resolutions for all downwind distances but the first one, and this is more evident for $x/\delta =3.75$. On the contrary, at the first downwind distance the numerical diffusion dominates and the 256 and 512 resolutions show low concentration values.

**Fig. 6:**
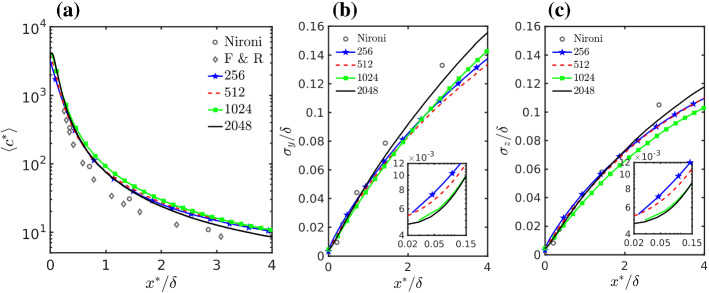
**a** Maximum mean concentration as a function of along-wind distance. Large-eddy simulation plume spread in **b** crosswind and **c** vertical directions. Insets in **b**, **c** are zoom over $0.02<x^*/\delta <0.15$

Figure 6a shows the scaled maximum mean concentration as a function of the equivalent along-wind distance from the source $x^*$ defined below. Here we include in the comparison the experimental measurements of Nironi et al. (2015) and F&R. As reported in Table 1, the simulations and the experiments have different mean velocity at the source; this in turn implies that the plume advection time at a certain distance from the source is different. Following Nironi et al. (2015), an appropriate dimensionless advection time can be defined accounting for different mean velocity at the source and different scaling parameter $u_*$ and $\delta$ as

6 $$\begin{aligned} T^{*}=\frac{x}{u_s}\frac{u_{*}}{\delta }. \end{aligned}$$

The comparison of plume results from different experiments should be done at the same dimensionless time, i.e., in our case $T_{(exp)}^{*}=T_{(LES)}^{*}$. This equality allows the definition of an equivalent dimensionless advection distance where the experimental results have the same dimensionless advection time as the LES results,

7 $$\begin{aligned} \frac{x_{(exp)}}{\delta _{(exp)}}=\frac{x_{(LES)}}{\delta _{(LES)}}\frac{u_{s(exp)}}{u_{s(LES)}} \frac{u_{*(LES)}}{u_{*(exp)}}. \end{aligned}$$

Based on this equality, we define the equivalent distance $x^*$ as

8 $$\begin{aligned} x^* = {\left\{ \begin{array}{ll} x,\,\,\,\,\,\,\,\,\,\,\,\,\,\,\,\,\,\,\,\,\,\,\,\,\,\,\,\,\,\,\,\,\,\,\,\,\,\,\, \text {LES} \\ x\frac{u_{s(LES)}}{u_{s(exp)}} \frac{u_{*(exp)}}{u_{*(LES)}}, \,\, \text {Wind-tunnel}\,\, \text {experiments}. \end{array}\right. } \end{aligned}$$

Similarly to the use of the dimensionless advection time, using this distance eliminates the difference originating simply by the mean advection velocity difference and allows also the comparison of plume dispersion from experiments with different $u_{*}$ and $\delta$. The LES results and the data of Nironi et al. (2015) have the same $u_{*}$ and $\delta$, so the scaling only differs through the mean velocities, $u_{s}$, but is limited since the difference in velocity is small. For $u_{s(LES)}$, needed in Eq. 8, the average value between the two most resolved simulations was used. We note that the LES results have not been corrected for the slightly different advection velocities as we want to underline the difference originating from grid resolution in the same exact simulation settings (the correction would be anyway minimal). In Fig. 6a the measurements of Nironi et al. (2015) and F&R have a source size that is smaller than (about half of) that used in the simulations, but source size has a very limited effect on mean concentration and can be neglected here. The model results are in quite good agreement with the measurements, especially those of Nironi et al. (2015), but the maximum mean concentration is generally overestimated and therefore plume spread must be underestimated. This is consistent with the lower simulated variances in the crosswind and vertical velocity components as shown in Fig. 1e, f. Figure 6b, c show the calculated crosswind and vertical plume spread standard deviation as a function of the distance from the source. These standard deviations are defined as the square root of the following variances

9a $$\begin{aligned} \sigma ^2_y(x) = \frac{\int \left\langle {\overline{c}}(\mathbf{x }) \right\rangle {(y- \left\langle y(x) \right\rangle )}^2 \, dydz }{\int \left\langle {\overline{c}}(\mathbf{x }) \right\rangle \, dydz}, \sigma ^2_z(x) = \frac{\int \left\langle {\overline{c}}(\mathbf{x }) \right\rangle {(z- \left\langle z(x) \right\rangle )}^2 \, dydz }{\int \left\langle {\overline{c}}(\mathbf{x }) \right\rangle \, dydz} \end{aligned}$$

with

9b $$\begin{aligned} \left\langle y(x) \right\rangle = \frac{\int \left\langle {\overline{c}}(\mathbf{x }) \right\rangle y \, dydz }{\int \left\langle {\overline{c}}(\mathbf{x }) \right\rangle \, dydz}, \left\langle z(x) \right\rangle = \frac{\int \left\langle {\overline{c}}(\mathbf{x }) \right\rangle z \, dydz }{\int \left\langle {\overline{c}}(\mathbf{x }) \right\rangle \, dydz} \end{aligned}$$

being the local time-averaged mean plume centre of mass coordinates. The variances $\sigma ^2_y$ and $\sigma ^2_z$ are also commonly called the absolute plume dispersion (e.g., Arya 1999; Dosio and de Arellano 2006). In the main panels of Fig. 6b, c, it is evident that the 2048 simulation has a larger averaged plume spread compared to the other simulations for $\sigma _i/\delta$ larger than about 0.06, and that the simulations have generally lower averaged plume spread compared to the wind-tunnel measurements apart from the closest location. The insets in 6b, c show the near source behaviour. In this case the two less resolved simulations have a higher spread and this is due to numerical diffusion as further discussed in Sect. 4.3.

To explain the mean concentration behaviour it is useful to recall an approximation of plume-spread standard deviation commonly used in conjunction with Gaussian dispersion models. Using the slender plume approximation (e.g., Arya 1999) and adapting Taylor’s dispersion theory (Taylor 1922) to anisotropic turbulence the evolution of the plume-spread standard deviation, in crosswind $(\sigma _y)$ and vertical $(\sigma _z)$ directions can be approximately written as

10a $$\begin{aligned} \sigma _{y}^2&= \sigma _{s}^2 + \sigma _v^2 T_{Lv} ^2 \left[ 2\frac{t}{T_{Lv}} - 2\left( 1-\mathrm {exp} \left( -\frac{t}{T_{Lv}}\right) \right) \right] \end{aligned}$$

10b $$\begin{aligned} \sigma _{z}^2&= \sigma _{s}^2 + \sigma _w^2 T_{Lw}^2 \left[ 2\frac{t}{T_{Lw}} - 2\left( 1-\mathrm {exp} \left( -\frac{t}{T_{Lw}}\right) \right) \right] , \end{aligned}$$

where $\sigma _{s}$ is the source standard deviation, $\sigma _v^2$ and $\sigma _w^2$ are the variances of the crosswind and vertical velocity components, and $T_{Lv}$ and $T_{Lw}$ are the crosswind and vertical Lagrangian integral time scales, respectively (e.g., Arya 1999). For an elevated plume this approximation is commonly used to describe plume dispersion by taking all the values as those at the source elevation and replacing time and downwind distance according to Taylor’s hypothesis $t=x / \left\langle {\overline{u}} \right\rangle$. These formulations remind us (e.g., Arya 1999) that for $t<<T_{L}$ turbulent dispersion is insensitive to $T_{L}$, while for $t>>T_{L}$ standard deviation of plume dispersion increases proportionally to $(T_{L}t)^{1/2}$. Close to the source the mean concentration is mainly influenced by the velocity variance, the mean plume increases as $\sigma _{y,z}^2 = \sigma _{s}^2 + \sigma _{v,w}^2 t^2$ (e.g., Arya 1999). As discussed above, the 2048 simulation has slightly greater variances $(\sigma _v^2, \sigma _w^2)$ but considerably larger structures (see Table 2) compared to lower resolutions. Larger structures result in longer correlation time scales including Lagrangian autocorrelations. For a neutral boundary-layer flow following Tennekes and Lumley (1972) the two-point length scales can be directly related to the Lagrangian integral time scales as $T_{Lv} \approx L_{vv,y}/\sigma _{v}$, $T_{Lw} \approx L_{ww,z}/\sigma _{w}$, $T_{Lu} \approx L_{uu,x}/\sigma _{u}$. Table 3 shows the Lagrangian time scale estimate according to Tennekes and Lumley (1972) and the estimates obtained by fitting Taylor’s dispersion relation (Eq. 10) to the plume spread in the crosswind and vertical directions. These are all rather crude estimates, especially in the vertical direction, but are adequate for cross comparing grid resolutions. The 2048 simulation has larger structures and accordingly longer Lagrangian time scales, especially in the crosswind direction, compared to the lower-resolution simulations, which leads to the lower maximum mean concentration of the scalar.

**Table 3:** Estimate of the Lagrangian time scales ($T_{lv}, T_{lw}$) from a fit of Taylor’s dispersion relations (Eq. 10) to the plume spread in crosswind and vertical directions for all grid resolutions. The estimates of the Lagrangian time scales as the ratio of length scales and standard deviations of velocity components following Tennekes and Lumley (1972) are also given

Resolution	$T_{Lv} (\text {s})$	$T_{Lw}(\text {s})$	$L_{uu,x}/\sigma _u(\text {s})$	$L_{vv,y}/\sigma _v(\text {s})$	$L_{ww,z}^{(+/-)}/\sigma _w(\text {s})$
$256 \times 64 \times 64$	0.15332	0.12187	0.755	0.347	0.450–0.324
$512 \times 128 \times 128$	0.14766	0.12217	1.950	0.301	0.338–0.247
$1024 \times 256 \times 256$	0.17305	0.10488	2.012	0.297	0.318–0.232
$2048 \times 512 \times 512$	0.20859	0.14121	2.525	0.417	0.374–0.236

### Concentration Fluctuations Variance and Budget

**Fig. 7:**
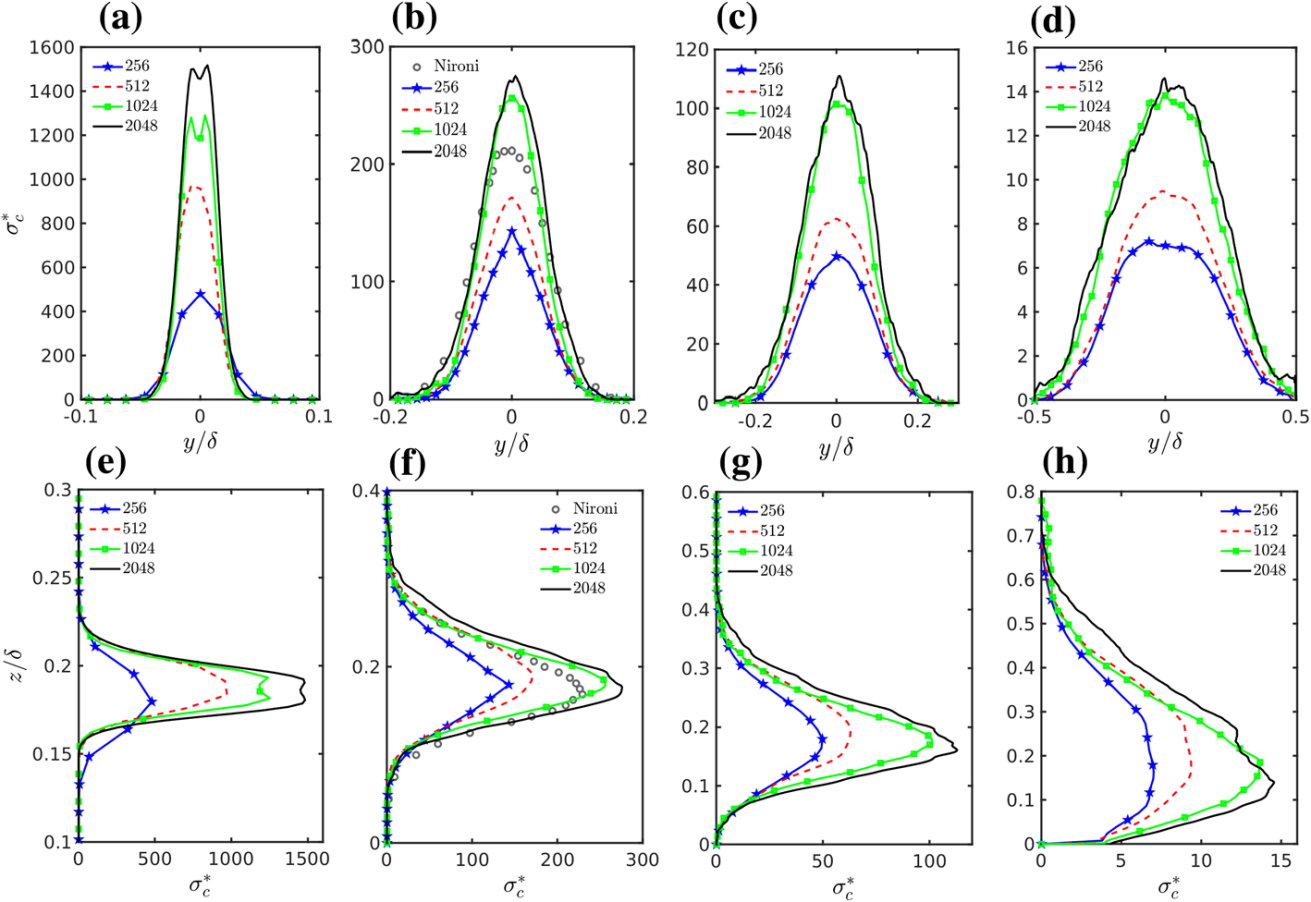
**a**–**d** Transversal, and **e**–**h** vertical profiles of concentration fluctuation standard deviation at various downwind distances, **a**, **e**$x^*/\delta =0.13$, **b**, **f**$x^*/\delta =0.725$, **c**, **g**$x^*/\delta =1.25$ and **d**, **h**$x^*/\delta =3.75$. Experimental data are from Nironi et al. (2015). Note that the source size of Nironi et al. (2015) data is $d_s = 7.5\times 10^{-3}\delta$

Figure 7 shows the crosswind and vertical profiles, passing through the position of maximum mean concentration, for the scaled standard deviation of concentration $\sigma _c^{*} = \sigma _c (u_s \delta ^2 / Q)$, $\sigma _c=(\langle {\bar{c}}''^2 \rangle )^{(1/2)}$. The effect of grid resolution on scalar fluctuations is evident in the standard deviation (Fig. 7). The change in the mesh resolution between the 256 and 1024 simulations does not produce significant change in the mean field while the scalar fluctuations are more than doubled. The increase is also substantial between the 512 and 1024 grid specifications. In contrast, 2048 and 1024 grid configurations seem to converge to quite similar results and are the only simulations able to capture the presence of the double peak at $x^*/\delta =0.13$. The behaviour in Fig. 7 can be summarized stating that the standard deviation converges at the 1024 grid configuration. This behaviour is in striking contrast with what was observed for the mean concentration where all the simulations but the 2048 one gave quite similar results. This is due to the fact that the mechanisms driving the evolution of the mean and the variance of the concentration are different and are differently influenced by the numerical resolution.

The budget analysis of the resolved scalar variance is introduced to highlight the relevant terms defining the observed concentration variance. The budget is calculated at the position of maximum mean concentration where most of the TKE is resolved for all resolutions. In a stationary state, the budget equation for the resolved scale mean scalar variance is written as

11 $$\begin{aligned} 0 = \underbrace{-\langle {\bar{u}}_i \rangle \frac{\partial \langle {\bar{c}}''^2\rangle }{\partial x_i}}_{\text {Adv.}} \underbrace{- 2\langle {\bar{u}}_i''{\bar{c}}''\rangle \frac{\partial \langle {\bar{c}}\rangle }{\partial x_i}}_{\text {Prod.}} \underbrace{-\frac{\partial \langle {\bar{u}}_i''{\bar{c}}''^2 \rangle }{\partial x_i}}_{\text {T.T.}} - \xi _{res} \end{aligned}$$

where the first term on the r.h.s. corresponds to the advection (Adv.), the second term corresponds to the production (Prod.), the third one corresponds to the turbulent transport (TT) and $\xi _{res}$ is the mean scalar dissipation from the residual. The definition of the scalar dissipation residual follows the same logic applied above to the resolved scale TKE budget and it is comprehensive of numerical dissipation. Two additional estimates of the mean scalar dissipation are possible: one is based on the equilibrium approximation for the SGS (Sykes and Henn 1992; Kaul et al. 2009; Heinze et al. 2015) and reads,

12 $$\begin{aligned} \xi _{eqm}= K_s \left\langle { \frac{\partial {\bar{c}}}{\partial x_i} \frac{\partial {\bar{c}}}{\partial x_i} } \right\rangle \ . \end{aligned}$$

**Fig. 8:**
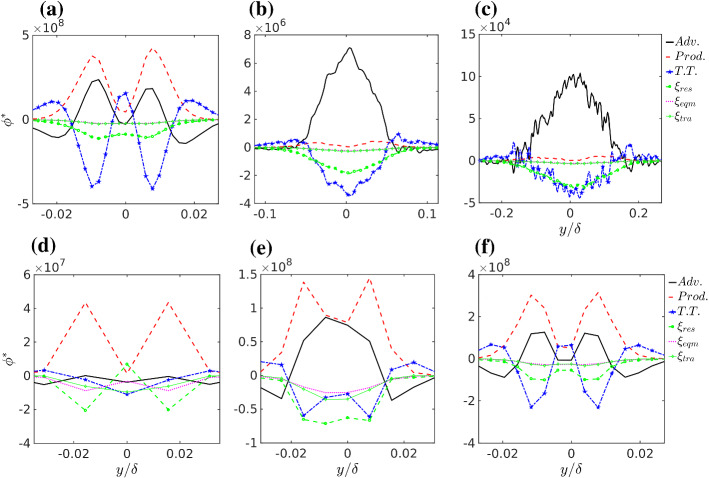
**a**–**c** Variance budget analysis of scalar concentration as a function of crosswind direction at different downwind distances, **a**$x^*/\delta =0.13$, **b**$x^*/\delta =0.725$, and **c**$x^*/\delta =1.92$, for 2048 simulation with $N_y \times N_z=512 \times 512$. **d**–**f** The same analysis but at $x^*/\delta =0.13$ for different grid resolutions, **d** 256 with $N_y \times N_z=64 \times 64$, **e** 512 with $N_y \times N_z=128 \times 128$ and **f** 1024 simulation with $N_y \times N_z=256 \times 256$

Alternatively the transfer of resolved-scale scalar variance to the SGS may be defined as (Heinze et al. 2015; Mironov and Sullivan 2016),

13 $$\begin{aligned} \xi _{tra} = \left\langle { \frac{\partial \tau _{ci}''}{\partial x_i} {\bar{c}}'' } \right\rangle \end{aligned}$$

where $\tau _{ci}=\overline{u_i'c'}$ is the SGS scalar flux computed by the SGS model. These two estimates have been calculated and considered in the analysis. However, they do not account for numerical dissipation. Therefore, similarly to what was discussed above for the TKE, they cannot be considered reliable. Figure 8a–c show the budget at the elevation of maximum mean concentration in the crosswind direction for the 2048 simulation. All the terms are indicated as $\phi$, and are reported normalized on the ordinate as $\phi ^* = \phi \frac{\delta }{u_{*}} \left( \frac{u_s \delta ^2}{Q} \right) ^2$. The production term, turbulent transport and advection are all important close to the source $(x / \delta = 0.13)$ but already at $x / \delta = 0.725$ the budget is dominated by advection and turbulent transport. At $x / \delta = 1.92$ advection dominates and dissipation $(\xi _{res})$ becomes as large as turbulent transport. This analysis agrees and completes what was found by F&R, that for distances $x / \delta > 1.92$ advection and dissipation dominates the balance. Figure 8d–f show the budget at $x / \delta = 0.13$ but for the grid resolutions 256, 512 and 1024. Production is the dominant term but for 256 and 512 simulations it has much lower values compared to the 2048 one (Fig. 8a). The shape of the terms is quite different in the 256 and 512 simulations compared to the 2048 one. In contrast, the 1024 simulation shows quite similar values and shape to those of the 2048 simulation. However, the production is still lower. This confirms that a tendency to converge is observed between 1024 and 2048 resolutions for the concentration variance, but the convergence is not yet perfect.

This analysis demonstrates that production close to the source is the critical phase for plume concentration fluctuations variance and that only an appropriate resolution can capture it. As the production of concentration variance occurs close to the source while afterward advection is the dominant term, we may conclude that significant converge, albeit not total in the concentration variance, is ensured by the source being resolved by at least $4^3$ grid nodes with the current numerical schemes. The next section analyzes the mechanism-generating fluctuations and how the insufficient resolution alters it.

### Absolute and Relative Dispersion, Meandering Motions, and Production of Fluctuations

Eddies of a size larger than the plume generate meandering fluctuations (Gifford 1959; Csanady 1973; Franzese and Cassiani 2007; Cassiani et al. 2009), i.e. large-scale undulations of the plume as a whole and the sweeping of the plume centreline with respect to the source location. By contrast, eddies of a size comparable to, but smaller than, the plume generate relative dispersion, that is the spreading of the plume in a coordinate system relative to the instantaneous centre of mass position (Richardson 1926; Batchelor 1952; Monin and Yaglom 1975; Sawford 2001; Franzese and Cassiani 2007). Eddies much smaller than the plume contribute little to relative dispersion (e.g., Mikkelsen et al. 1987).

When assuming that the associated spatial scales are separated, the absolute dispersion of the plume can be partitioned between two statistically independent components (Gifford 1959; Csanady 1973; Franzese and Cassiani 2007). Therefore the absolute dispersion defined in (9a), can also be written as

14 $$\begin{aligned} \sigma ^2_y = \sigma ^2_{my} + \sigma ^2_{ry}\,\,\,\,\,\, ,\,\,\,\,\,\, \sigma ^2_z = \sigma ^2_{mz} + \sigma ^2_{rz} . \end{aligned}$$

The meandering variance can be readily defined from the local centre-of-mass definition,

15 $$\begin{aligned} y_{m}(x) = \frac{\int {\overline{c}}(\mathbf{x }) \, y \, dydz }{\int {\overline{c}}(\mathbf{x }) \, dydz}, z_{m}(x) = \frac{\int {\overline{c}}(\mathbf{x }) \, z \, dydz }{\int {\overline{c}}(\mathbf{x }) \, dydz}, \end{aligned}$$

as

16 $$\begin{aligned} \sigma ^2_{my} = \left\langle y_{m}^2 \right\rangle - {\left\langle y_{m}\right\rangle }^2 \,\,\,\,\,\, ,\,\,\,\,\,\, \sigma ^2_{mz} = \left\langle z_{m}^2 \right\rangle - {\left\langle z_{m}\right\rangle }^2 . \end{aligned}$$

**Fig. 9:**
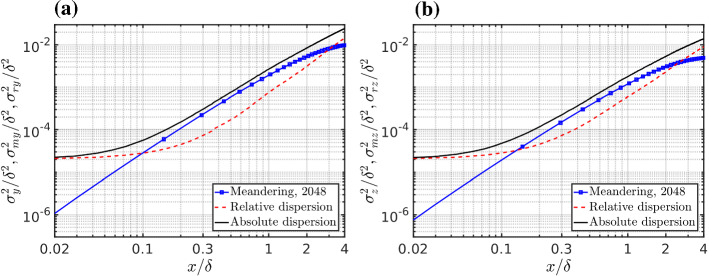
Absolute and relative dispersion, and the meandering motion of the plume, as a function of downwind distance for the 2048 simulation for, **a** crosswind, and **b** vertical directions

**Fig. 10:**
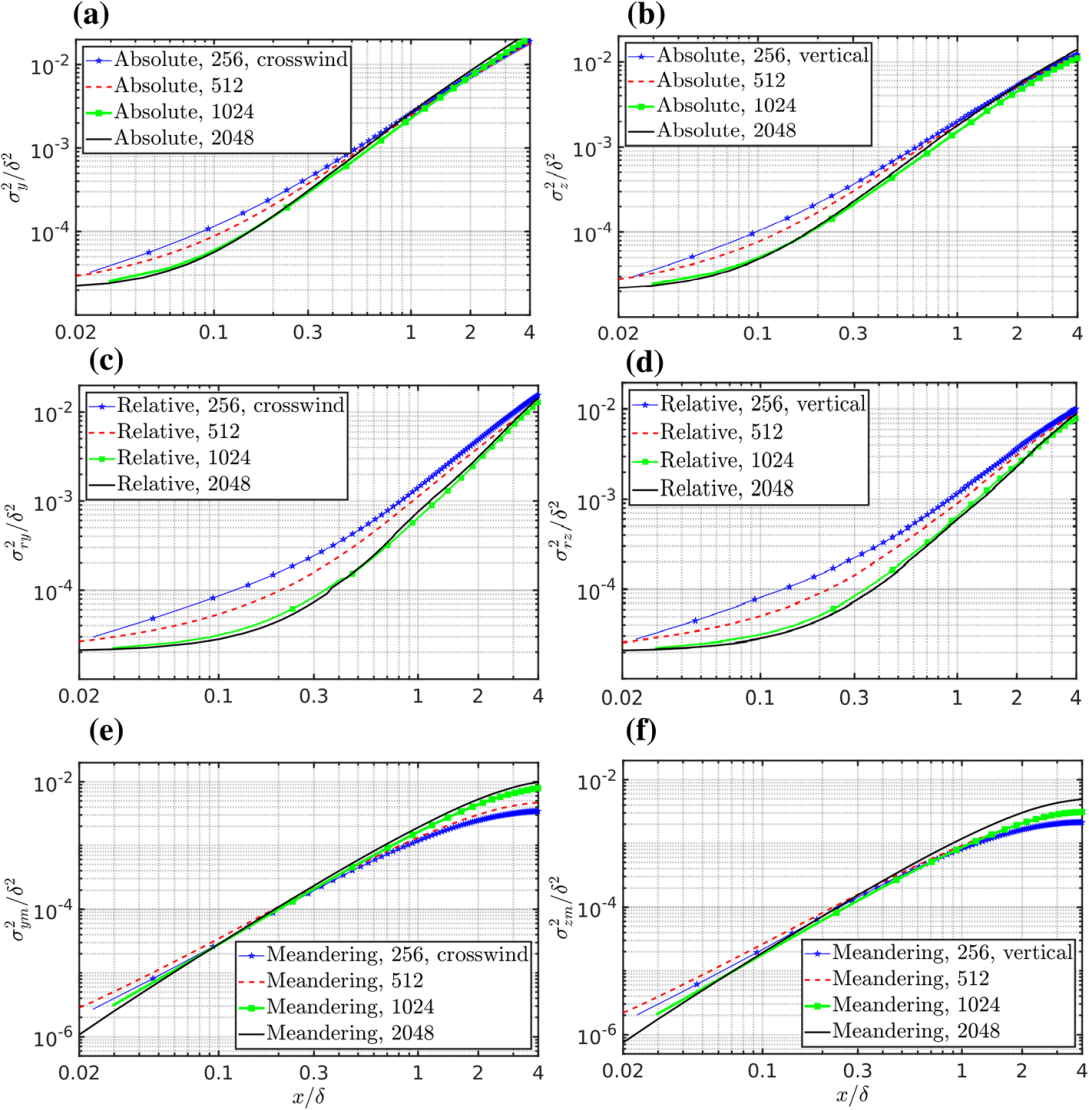
Comparison of **a**, **b** the absolute, **c**, **d** the relative dispersion, and **e**, **f** meandering motion among the different grid resolutions. Left panels **a**, **c**, **e** are for the crosswind direction and right panels **b**, **d**, **f** are for the vertical directions

The relative dispersion can then be calculated from Eq. 14. Absolute dispersion, relative dispersion and meandering, as a function of along-wind distance from the source, are reported in Fig. 9 for the 2048 simulation. Figure 10 represents the comparison of the absolute and relative dispersion between different grid resolutions. At a constant absolute dispersion, the smaller the relative dispersion is, the higher the fluctuations are since the meandering related production of fluctuations is enhanced by two mechanisms, the increased flapping of the plume and the fact that a smaller and more concentrated plume is moving. This is most important very close to the source, as illustrated in Fig. 10c, d, where most of the production of the scalar variance occurs, as extensively discussed above using the variance budget. Therefore we may hypothesize that a coarse grid resolution increases the relative dispersion by numerical diffusion and artificially damps the meandering production of concentration fluctuations in a way similar to an increase in the source size and this decreases the production of scalar variance. After this initial phase the scalar variance is mainly transported away and eventually dissipated far away from the source.

The hypotheses discussed above can be nicely and quantitatively demonstrated by using the meandering plume model of Gifford (1959) (see Appendix 3 for more details). In this model the concentration moments can be obtained from $\sigma ^2_m$ and $\sigma ^2_r$, which, in the present case, are those directly obtained from the LES concentration field with no parametrization used. The meandering plume model shows that the production of fluctuations by meandering depends on the ratio $M=\sigma ^2_m/\sigma ^2_r$ and on the value of $\sigma ^2_r$ (see Appendix 3), larger $\sigma ^2_r$ and smaller *M* generate a decrease in fluctuations.

**Fig. 11:**
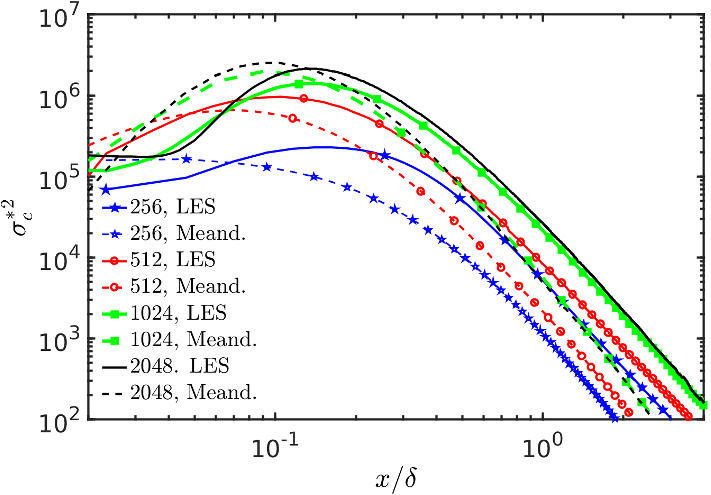
Comparison of the scalar variance of LES and the meandering plume model. Dashed lines represent the meandering plume model on the centreline while the solid lines correspond to the LES results in the position of maximum mean concentration. All the quantities in the meandering plume model (Eq. 28 in Appendix 3) are derived from the LES concentration field with no parametrization

The second moment of the concentration from LES and from the meandering plume model are compared in Fig. 11 as a function of downwind distance from the source. Dashed lines correspond to the meandering plume model and solid lines correspond to the LES results at various grid resolutions and at the position of the peak of mean concentration. The meandering plume model reproduces well the value of the variance peak generated by the LES concentration time series at different resolutions. Increasing values are observed by increasing resolution both for the meandering model and for the LES. The agreement is better at the highest resolution. The position of the peak in the meandering is slightly displaced compared to the LES but this difference decreases with resolution. Further downwind, as expected, a considerable difference arises between the meandering plume model and the LES because in the fluctuating plume formulation used here any contribution of internal fluctuations, in the coordinate system relative to the local plume centre of mass, is neglected.

The meandering results in Fig. 11 prove the physical picture discussed above showing that the production of fluctuations at coarse resolutions decreases because of artificially enhanced relative dispersion by numerical diffusion. It also shows that the meandering production of fluctuations for the 1024 and 2048 simulations is similar, thus supporting the existence of a convergence of LES results for the concentration fluctuations variance between the 1024 and 2048 resolutions.

### Scaled Concentration Moments and Probability Density Function

So far the mean concentration and the variance have been analyzed, and we will now focus on the shape of the concentration p.d.f.. A parameter that is often used to characterize the p.d.f. shape is the relative intensity of concentration fluctuations (see e.g. F&R) defined as $i_c = \sigma _c / \left\langle {\overline{c}} \right\rangle$. This is also called the coefficient of variation in statistical textbooks, and describes the spread of the p.d.f. relative to its mean value. As discussed in, e.g., Yee et al. (1993c), Nironi et al. (2015), Marro et al. (2018) $i_c$ suffices to define the shape of the Gamma p.d.f., which is often used as an analytical model of the concentration p.d.f. based on experimental evidences and theoretical arguments on scalar mixing in confined domains (Duplat and Villermaux 2008). So far it was observed that the $N_x=2048$ simulation has different flow structures, with larger $T_L$, which generates larger mean dispersion and lower maximum mean concentration. The concentration variance instead was found to approach convergence only for $(N_x)\ge 1024$.

Following F&R and Nironi et al. (2015), Fig. 12 shows the intensity of concentration fluctuations defined as $\frac{\max (\sigma _c(x))}{\max (\left\langle \overline{c(x)} \right\rangle )}$ as a function of along-wind distance from the source. Note that these two maxima are not necessarily in the same position, and that the ratio increases systematically with increasing of the resolution. However, following the results above on mean concentration and variance, it is clear that the changes in the relative intensity of the concentration fluctuations moving from 256 to 512 resolutions and from 512 to 1024 resolutions is dominated by an increase in the fluctuations, i.e. $\sigma _c$. On the contrary, the increase in intensity from 1024 to 2048 resolutions is dominated by the change (decrease) of mean concentration ($\left\langle {\overline{c}} \right\rangle$) while the standard deviations of fluctuations are very similar between the two resolutions.

Overall, $i_c$ does not tend to converge as nicely as the standard deviation when moving from the 1024 to 2048 resolution, with a relative difference of about 30% over most of the distances. However, the fractional variation between 512 and 1024 simulations is clearly more pronounced than between 1024 and 2048 simulations.

**Fig. 12:**
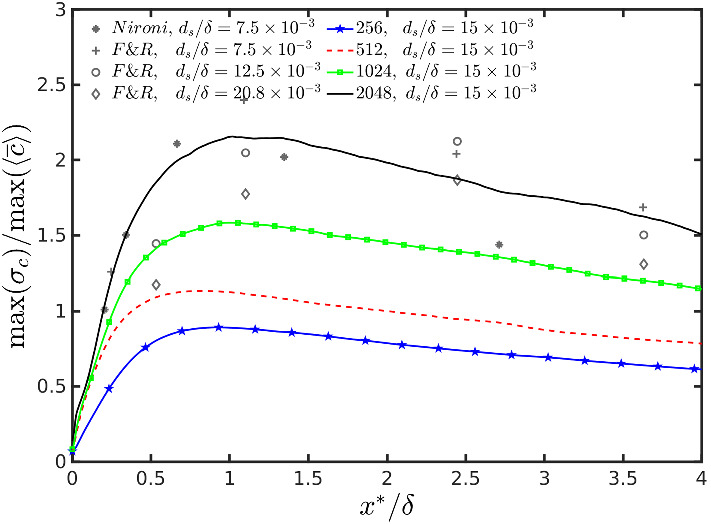
Intensity of concentration fluctuations $\frac{\max (\sigma _c)}{\max (\langle {\overline{c}}\rangle )}$ for different grid resolutions as a function of downwind position, and comparison with Nironi et al. (2015) and Fackrell and Robins (1982) experimental data for several source sizes

The comparison with the wind-tunnel experiments in Fig. 12 shows that the 2048 simulation is in better agreement with the measurements. We note that the source sizes are not identical but our source size lies in between the Nironi et al. (2015) and F&R configurations. A remarkable point is that the relative intensity of fluctuations in the LES seems to have a slower decay compared to the wind-tunnel observations. This indicates that, despite SGS and numerical diffusivity and despite neglecting SGS fluctuations, the LES has less mixing and lower dissipation.

**Fig. 13:**
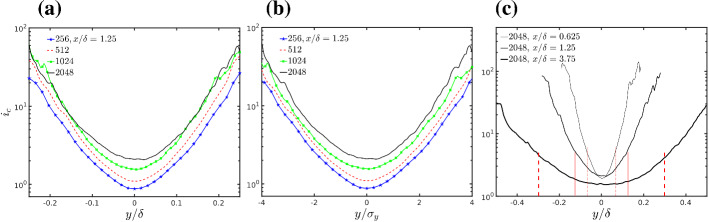
**a** Intensity of concentration fluctuation ($i_c = \frac{\sigma _c}{\langle {\overline{c}} \rangle }$) in the crosswind direction at position of maximum mean concentration and $x/\delta = 1.25$ for all resolutions; **b** same as panel (a) but plotted as a function of $y/\sigma _y$; **c** intensity of concentration fluctuation in crosswind direction for 2048 simulation at different downwind distances from the source. The vertical lines correspond to $\pm 2 \sigma _y$ at the various downwind distances

Figure 13a shows the crosswind profile of $i_c$ at the downwind distance $x / \delta =1.25$ and elevation of maximum mean concentration for all the mesh refinements. The centreline $(y / \delta =0)$ is a point of minimum in the intensity of concentration fluctuations. Apparently, the lines become progressively closer to the crosswind distance and for $\left| y\right| / \delta \ge 0.1$ the values of 1024 and 2048 simulations are almost indistinguishable. However, this is an effect of the different plume spreads. Figure 13b shows that normalizing the crosswind coordinate by the local standard deviation of plume spread, $\sigma _y$, the curves become similarly spaced in the centreline and towards the plume edges. Figure 13c shows $i_c$ for the 2048 simulation, as a function of the crosswind position and for three downwind distances from the source. The vertical dashed lines mark distances of $2 \sigma _y$ from the plume centreline on each side and indicate the position of the plume edges.

The skewness $Sk={\left\langle \left( {\bar{c}}-\left\langle {\bar{c}} \right\rangle \right) ^3 \right\rangle } / \sigma _c^{3}$ and kurtosis $Ku={\left\langle \left( {\bar{c}}-\left\langle {\bar{c}} \right\rangle \right) ^4 \right\rangle } / \sigma _c^{4}$ are commonly used indicators of p.d.f. shape. The skewness measures the degree of asymmetry of the p.d.f. and the kurtosis indicates the significance of the tails of the p.d.f.. However, the concentration is bounded at zero, $c\ge 0$, and the p.d.f. is positively skewed over most of the downwind distances. Therefore, *Sk* and *Ku* are both strongly influenced by the right tail of the p.d.f..

**Fig. 14:**
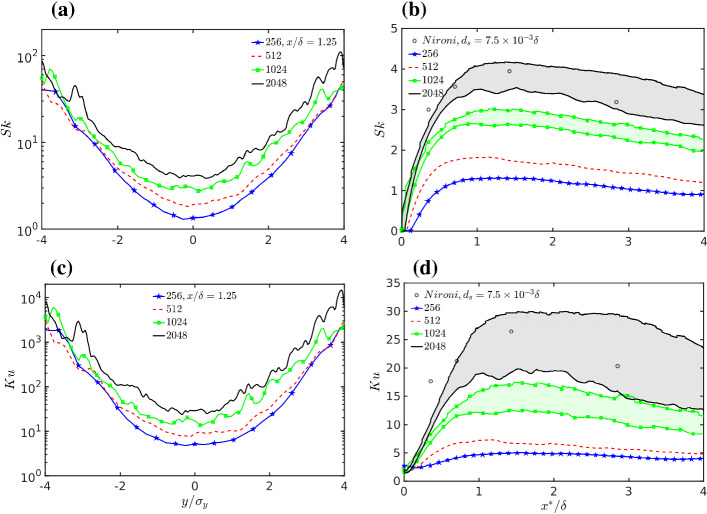
**a** Crosswind variation of skewness at elevation of maximum mean concentration for $x / \delta =1.25$; **b** along-wind variation of skewness on the position of maximum mean concentration. For the 1024 and 2048 simulations, the lower curves are the minimum in a small area surrounding the mean concentration maximum, while the upper curves correspond to the average over the same area used for the minimum. For 256 and 512 simulations, the two estimates are very similar and only the minimum is reported. See text for more details. **c**, **d** As in **a**, **b** but for the kurtosis

Figures 14a, c show the crosswind variation of *Sk* and *Ku* at an elevation of maximum mean concentration, for $x / \delta =1.25$ and all grid resolutions, plotted as a function of $y / \sigma _y$ to avoid overlapping due to the differences in mean plume spread. Both *Sk* and *Ku* strongly increase while moving towards the plume edges where the concentration is more intermittent. Moving towards the plume edges, it seems that the relative difference found in the centreline between grid resolutions disappears between 256 and 512 resolutions while it is conserved between 1024 and 2048 resolutions. However, despite the long averaging time, these high-order statistics are subject to large statistical fluctuations for both 1024 and 2048 resolutions. The statistical fluctuations make the interpretation of the data difficult, especially in the plume edges. However, according to the Gamma p.d.f. (Eq. 17) model $Sk=2i_c\approx 100$ for our dataset in the plume edges, and $Ku=1.5 Sk^2+3 \approx 15000$, this seems to suggest that the values reported are not far from reality.

Figures 14b, d show the along-wind variation of *Sk* and *Ku* on the position of maximum mean concentration. More precisely, the minimum of the statistics over a small area $(\Updelta y \times \Updelta z)$ surrounding the position of maximum mean concentration is reported together with the spatially-averaged value over the same area. The area starts with source size and afterward expands up to $\Updelta y =\Updelta z = 2 \times 0.037 \delta$ to account for the plume expansion. This approach was used because the value exactly in the position of maximum was affected by fluctuations that hindered the detection of the along-wind variation. The two estimates are respectively biased towards low values (the minimum over the area) and towards high values (the average over the area). For the average, the bias arises from the crosswind profile strongly increasing away from the plume centre. Together, the two curves provide an upper and lower bound and give a good indication of the behaviour of the statistics along the plume centreline. For the lower resolutions, i.e., the 256 and 512 simulations, only one line (the minimum) is reported because the two estimates are very similar.

On the plume centreline, a significant increase is visible for both *Sk* and *Ku* while increasing the resolution, and is similar to the behaviour of $i_c$ but more pronounced. This means that the weight of the right tail in the p.d.f. increases with the grid resolution. However, a careful inspection of Fig. 14b reveals that, for *Sk*, the relative difference between the 2048 and 1024 simulations is about $50\%$ and much lower than between 1024 and 512, about $100\%$. This suggests that also for *Sk* a clear tendency to convergence is observed between the 1024 and 2048 simulations. It is remarkable that the difference between 256 and 512 is rather small, highlighting again that LES-grid sensitivity studies must span a wide range of resolutions to be meaningful. Understanding the actual behaviour of *Ku* is more difficult since this statistic is affected by the largest statistical errors. Considering only the lower bound of the estimate (the curve given by the minimum in Fig. 14d) a tendency to converge could be again deduced between the 1024 and 2048 simulations, but this is not the case considering the average over the area, i.e. the upper bounds of the shaded area.

Overall, these results show that the behaviour of the right tail of the p.d.f. is even more sensitive to the changes of resolution than is the variance. This was perhaps expected but could be clearly appreciated only when the grid resolution moved from 512 to 1024 and 2048. In contrast, a change between 256 and 512 resolutions showed very limited change in *Sk* and *Ku* and would erroneously point towards an almost perfect convergence, while both these resolutions are clearly incapable of correctly capturing *Sk* and *Ku* and therefore the tail of the p.d.f..

A significant point is that the 1024 and 2048 simulations have very similar standard deviations of concentration over most of the downwind distances. Therefore, similar *Sk* and *Ku* values imply similar third- and fourth-order centred moments. This implies very similar actual concentration values in the p.d.f. tail considering that the differences are greatly enhanced for the third and fourth moment of concentration. Kurtosis especially highlights even quite limited discrepancies in the p.d.f. tail between the 1024 and 2048 resolutions.

Despite the different source sizes in the wind-tunnel experiment and LES, the comparison with the experimental values of Nironi et al. (2015) (see also Marro et al. 2018) shows that the values of both *Sk* and *Ku* for the 2048 simulation are reasonable and realistic. However, considering the larger source size in the LES, the upper bound of the LES estimates is somewhat higher than the experimental observations while the lower bound is in a good agreement with the observations.

As is evident from the changes in $i_c$, *Sk* and *Ku*, the shape of the p.d.f. significantly changes with the distance from the source, both in the along-wind and crosswind directions. This is true for any grid resolution.

**Fig. 15:**
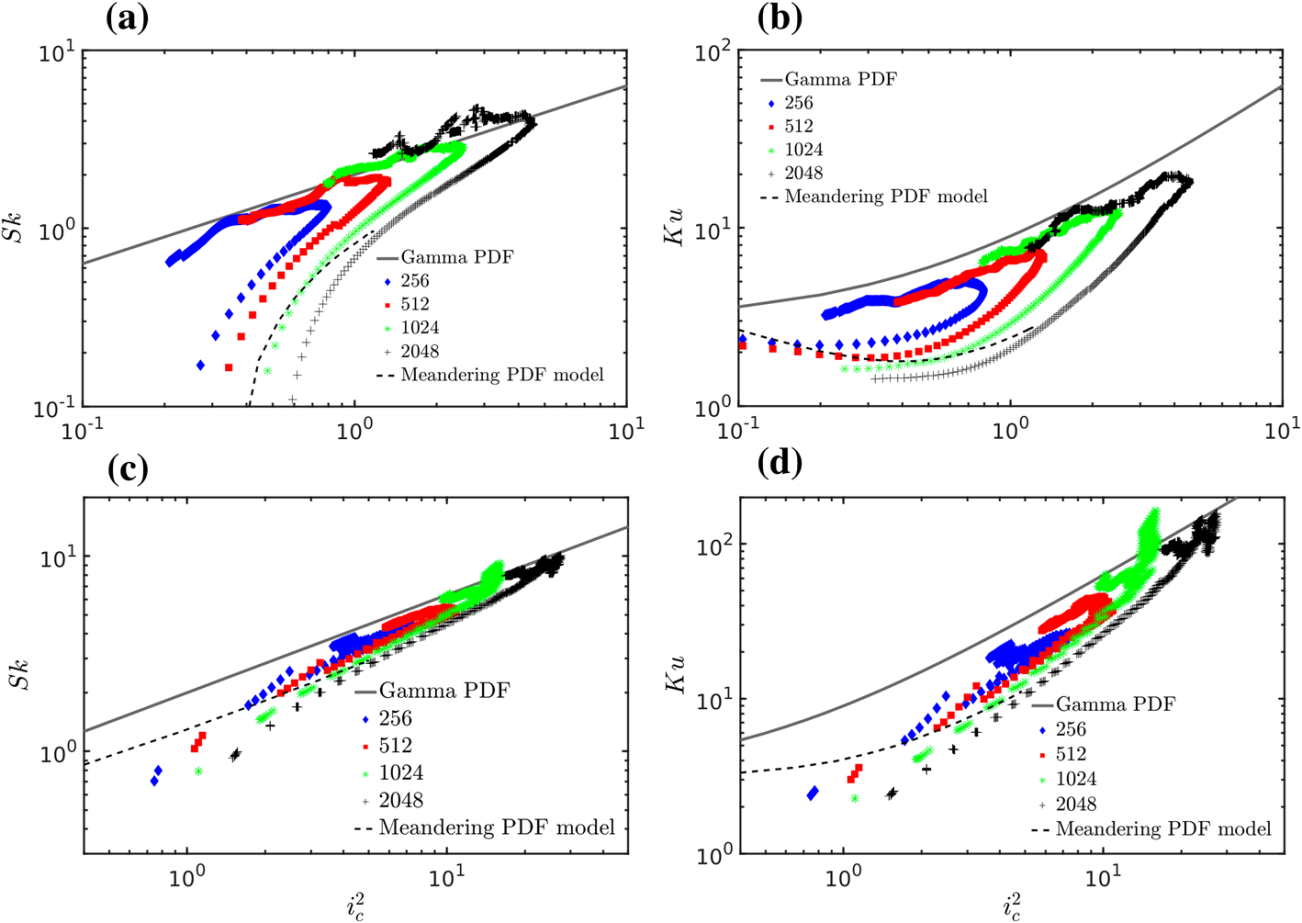
Minimum values of, **a** Skewness, and **b** kurtosis in the small area surrounding the maximum mean concentration as a function of $i_c^2$ where $i_c$ is the intensity of concentration fluctuations. **c** Skewness and **d** kurtosis at $2\sigma _y$ from the maximum mean concentration as a function of $i_c^2$

An analytical model of the concentration p.d.f. that in recent years has gained considerable experimental and theoretical support is a family of one parameter Gamma distributions (Duplat and Villermaux 2008; Yee and Skvortsov 2011; Nironi et al. 2015). The concentration p.d.f. is defined as

17 $$\begin{aligned} p(\chi ) = \frac{\kappa ^\kappa }{{\varGamma }(\kappa )} \chi ^{\kappa -1}\mathrm {exp}(-\kappa \chi ) \end{aligned}$$

with ${\varGamma }(\kappa )$ the Gamma function, $\kappa =i_c^{-2}$ and $\chi =c/\langle c\rangle$. For the Gamma p.d.f. $Sk=2 i_c$ and $Ku= 1.5 Sk^2 +3$ (e.g., Nironi et al. 2015).

Complementary to the Gamma p.d.f. model is the meandering plume model, extensively discussed above, which fully describes the concentration p.d.f. in the early phase of dispersion where concentration fluctuations are produced. According to the meandering plume model, the centred moments, skewness and kurtosis can be calculated using a standard relationship between centred and uncentred moments (e.g., Monin and Yaglom 1971). This (see Appendix 3, Eq. 28) highlights that close to the source and along the plume centreline ($y=y_s, z=z_s$), scaled moments such as $i_c$, *Sk* and *Ku* are only a function of $M_y=\sigma _{my}^2 / \sigma _{ry}^2$ and $M_z=\sigma _{mz}^2 / \sigma _{rz}^2$. Therefore plotting *Sk* and *Ku* as a function of $i_c$ gives a unique curve.

Figures 15a, b show the minumum values of skewness and kurtosis in the small area surrounding the maximum mean concentration for all grid resolutions as a function of $i_c^2$. The LES data show a clear path, with data moving from the meandering p.d.f. limit, close to the source (dashed line) with low values of $i_c$ progressively increasing toward the peak, to the Gamma p.d.f. far away from the source (solid line) with progressively decreasing value of $i_c$ after its peak. The peak in $i_c$ thus marks the start of the phase where the Gamma p.d.f. is the optimal model. This was never discussed before to our knowledge.

We note that the meandering plume model values are only shown for the increasing value of $i_c$ up to its peak (see also Fig. 11). Different resolutions seem to behave slightly different with the higher resolutions more closely following both the meandering model close to the source and the Gamma p.d.f. after the $i_c$ peak. This confirms that by increasing the resolution, the LES better captures the physics of dispersion.

Figure 15c, d report similar comparison but for the off centreline position $y=2 \sigma _y$. The meandering plume model values almost perfectly collapse on a single curve, therefore only one line is reported in Fig. 15c, d. It overall confirms the behaviour observed on the centreline but noticeably, at constant $i_c$, the difference between values of *Sk* and *Ku* in the meandering and dissipative phases is strongly reduced.

## Summary and Discussion

Four different grid resolutions have been used to perform LES of plume dispersion and concentration fluctuations in an incompressible half-channel flow at infinite Reynolds number. The analysis of the difference in results for changing resolution was supported by a comparison with wind-tunnel experimental data.

For the velocity field, the most salient points of the comparison among resolutions were that, despite the good consistency of one-point second-order velocity statistics, the two-point correlation analysis showed that the highest resolution simulation (2048) developed larger turbulent structures, characterized by longer length scales and time scales. The TKE spectrum revealed that an inertial subrange was present in the two more resolved simulations.

The comparison with the wind-tunnel data showed that the variance of the along-wind velocity component and TKE were well captured, especially at high resolutions, while the variance of crosswind and vertical components (which are more important for plume dispersion) was lower in the LES than in the measurements. The LES showed also a generally higher mean flow speed at source elevation compared to the experimental data of Nironi et al. (2015) and this could be mainly attributed to the specific wall model used in PALM.

Moving to the scalar field, the comparison of the mean concentration among the different resolutions showed that the highest resolution simulation (2048) had a markedly different mean field characterized by higher plume spread and lower mean concentrations. This was tracked back to a larger $T_L$ resulting from the larger turbulent structures generated at this grid resolution. As a result, the absolute dispersion obtained at this resolution was more similar to the wind-tunnel measurements despite still being underestimated.

The analysis of concentration variance among different resolutions showed a clear tendency to converge in the two higher resolved simulations (1024 and 2048). The analysis demonstrated that production close to the source is the most critical phase for plume concentration fluctuations and that only an appropriate resolution can capture it, corresponding to $N_z, N_y \ge 256$ and $N_x \ge 1024$ here. More precisely, the conservation equation of scalar variance showed that the production of fluctuations mainly occured close to the source while afterward advection was the dominant term. Therefore, significant convergence in the variance was ensured by the source being adequately resolved by at least $4^3$ grid nodes.

The mechanism of generating fluctuations was analyzed by separating plume dispersion in meandering and relative dispersion and using the Gifford (1959) meandering model for a quantitative analysis. This analysis demonstrated that inadequate resolution artificially enhanced relative dispersion, therefore suppressing meandering related production of fluctuations. As the meandering is often thought to be associated with the large eddies, one could think that a relatively low resolution LES is sufficient to capture it correctly. This intuitive picture is not correct and, as demonstrated in our analysis, numerical diffusion can suppress fluctuations produced by the meandering close to the source if the source itself is not properly resolved. This early meandering phase turns out to be very important for the production of concentration variance. The artificial diffusion imposed by a coarse grid resolution seems to result in a larger effective source size with the corresponding lower level of fluctuations.

The analysis of the shape of the p.d.f. included the examination of scaled moments up to the fourth, $i_c$, *Sk*, *Ku*. Regarding $i_c$, by comparing all the grid resolutions, the combined behaviour of mean concentration and standard deviation generated a weaker convergence of this scaled moment although a tendency to converge was again visible between the 1024 and 2048 simulations. However, two distinct reasons of this behaviour could be determined: (i) an increase in concentration variance production acting mainly on all resolutions up to 1024, and (ii) a decrease in the mean concentration acting on the 2048 resolution. Therefore, insufficient scalar source definition acted mainly on the 256 and 512 resolutions totally preventing $i_c$ convergence at these resolutions, while a change in the turbulent structure modified the mean value of the 2048 simulation.

Regarding *Sk* and *Ku*, some tendency to converge was again visible between the 1024 and 2048 simulations, especially for *Sk*. From 256 to 512 to 1024 resolutions, the production of fluctuations close to the source increases significantly. Here, the third and fourth centred moments increase even more than $\sigma _c$, because the plume retains a progressively thinner initial structure that afterward is transported by mean advection and turbulence; significantly different internal plume structure is present at different resolutions. Moving from 1024 to 2048 resolution the production of concentration variance is very similar and therefore the internal plume structure is similar over most of the concentration values. However the third and, more markedly, the fourth centred moments are mostly influenced by the higher concentration values in the right tail of the p.d.f. and strongly enhance even limited differences in the actual concentrations.

Irrespective of grid resolution, we found that the different p.d.f. shapes could be very nicely reproduced by a family of Gamma distribution in the decaying phase of $i_c$, i.e. downwind of its peak. While the use and observation of the Gamma p.d.f. is not new in the literature of plume dispersion (e.g Yee et al. 1993c; Yee and Skvortsov 2011; Nironi et al. 2015), this very clear transition of the concentration p.d.f. to a family of Gamma p.d.f. after the peak of $i_c$ was not observed before to our knowledge. We emphasize that a similar behaviour was observed here in the plume centre and in the plume periphery. The fact that all LES resolutions showed similar behaviour, irrespective of the quite different turbulent velocity spectrum, indicates that the mechanism generating the Gamma p.d.f. is rather general and well captured by the LES.

Complementary to the Gamma p.d.f. model is the concentration p.d.f. generated by the meandering plume model of Gifford (1959), which was shown to quite accurately capture the concentration p.d.f. generated by the LES in the early phase of plume dispersion, before the peak of $i_c$. This is especially the case for the higher resolutions, 1024 and 2048.

We underline that our study highlighted once more that convergence in the turbulent flow of LES is difficult to achieve and this is exacerbated for the scalar field from a localized small source. Different mechanisms drive the production of fluctuations and the long term evolution of mean plume dispersion and therefore multiple distinct factors could prevent convergence of the concentration p.d.f. shape. However, we systematically investigated the differences in the results among grid resolutions and discussed the mechanisms producing these differences. This provides a useful reference to anybody interested in validating and using LES for plume dispersion and concentration fluctuations at very high Reynolds number, and provides a quantification of the uncertainty involved in this type of studies. A very important point is that the grid dependency quantification should span a wide range of grid refinements, in our case the difference in scalar fluctuations between 256 and 512 resolutions was minimal while there was a large change between 512 and 1024 resolutions. Furthermore, the results clearly show that p.d.f. shapes could be very nicely reproduced by a family of Gamma distributions only in the decaying phase of $i_c$, downwind of its peak. The meandering p.d.f. was a better model in the early phase of dispersion.

Detailed analysis and comparison with wind-tunnel measurements showed that, as expected, the 2048 simulation produced the more realistic turbulent structures and therefore concentration p.d.f. evolution. Simulating a wind-tunnel flow means that some important characteristics of the real atmosphere are neglected in the simulations, like for example the momentum flux and TKE shear production at the boundary-layer top. However, the scalar source considered here is placed in the lower part of the boundary layer where these effects should be negligible compared to the interaction with the wall. It remains a task for future studies to elucidate any difference in concentration fluctuations between the current wind-tunnel setting and a full atmospheric boundary-layer simulation.

Finally, we remark that our results are somewhat specific to our set-up, in particular the SGS model, wall model and numerical methods. Many models are available and the convergence of the model results and the turbulence statistics may depend on the SGS model (e.g., Salesky et al. 2017) and wall model (e.g., Hultmark et al. 2013). In particular, it is likely that the use of suitably formulated Lagrangian stochastic SGS models for the scalar field may improve the convergence of the scalar field results and will be further investigated in future studies. However, the methodology presented here to validate and understand LES results for plume dispersion and concentration fluctuations is general and should be a useful guidance for anyone interested in using LES for such tasks.

## Appendix 1: Governing Equations

The equations for the conservation of mass, momentum and passive scalar, filtered over a grid volume on a cartesian grid, in PALM (Maronga et al. 2015) are

18a $$\begin{aligned} \frac{\partial {{\overline{u}}}_j}{\partial x_j}&= 0 \end{aligned}$$

18b $$\begin{aligned} \frac{\partial {{\overline{u}}}_i}{\partial t}&= - \frac{\partial {{\overline{u}}}_i {{\overline{u}}}_j}{\partial x_j} - \frac{1}{\rho _0}\frac{\partial \overline{\pi ^*}}{\partial x_i} - \frac{\partial }{\partial x_j}\left( \overline{u'_i u'_j}-\frac{2}{3}e\delta _{ij} \right) \end{aligned}$$

18c $$\begin{aligned} \frac{\partial {\overline{c}}}{\partial t}&= -\frac{\partial ({\overline{u}}_i {\overline{c}})}{\partial x_i} - \frac{\partial }{\partial x_i}\left( \overline{u'_i c'} \right) \end{aligned}$$

where $\pi ^* = p + 2/3 \rho _0e$ is the modified perturbation pressure with *p* being the perturbation pressure, $\rho _0=1\, {\text {kg m}}^{-3}$ is the density of dry air at the surface, and $e = \frac{1}{2} \overline{u_i'u_i'}$ is the SGS TKE. The SGS terms are parametrized using a 1.5-order closure after Deardorff (1980). The PALM code uses the modified version of Moeng and Wyngaard (1988) and Saiki et al. (2000) as

19a $$\begin{aligned} \overline{u'_i u'_j}-\frac{2}{3}e\delta _{ij}&= -K_m\left( \frac{\partial {{\overline{u}}}_i}{\partial x_j}+\frac{\partial {{\overline{u}}}_j}{\partial x_i} \right) , \end{aligned}$$

19b $$\begin{aligned} \overline{u'_i c'}&= -K_s \frac{\partial {\overline{c}}}{\partial x_i}, \end{aligned}$$

where $K_m = c_ml\sqrt{e}$ is the local SGS diffusivity of momentum, $c_m=0.1$ is a model constant, $l=\mathrm {min}(1.8z, \Updelta )$ is the SGS mixing length, and $\Updelta =\root 3 \of {\Updelta x \Updelta y \Updelta z}$ with $\Updelta x, \Updelta y, \Updelta z$ being the grid spacing in the $x,\, y$ and *z* directions, respectively. The SGS scalar diffusivity is defined as $K_s=\left( 1+\frac{2l}{\Updelta } \right) K_m$. Moreover the closure includes a prognostic equation for the SGS TKE,

20 $$\begin{aligned} \frac{\partial e}{\partial t} = - {{\overline{u}}}_i \frac{\partial e}{\partial x_i} - \left( \overline{u'_iu'_j} \right) \frac{\partial {{\overline{u}}}_j}{\partial x_i} -\frac{\partial }{\partial x_i}\left[ \overline{u'_i \left( e+\frac{p'}{\rho _0} \right) } \right] - \epsilon , \end{aligned}$$

in which the pressure term and $\epsilon$ are parametrized as

21a $$\begin{aligned} \left[ \overline{u'_i \left( e+\frac{p'}{\rho _0} \right) } \right]&= -2K_m\frac{\partial e}{\partial x_i}, \end{aligned}$$

21b $$\begin{aligned} \epsilon&= \left( 0.19+0.74\frac{l}{\Updelta }\right) \frac{e^{3/2}}{l}. \end{aligned}$$

## Appendix 2: Boundary Conditions

A variety of boundary conditions are available in PALM. In the current study, for the velocity field a periodic boundary condition is used in along-wind (*x*) and crosswind (*y*) directions. At the bottom wall, the lowest grid level for horizontal velocity components and scalar quantities is not staggered vertically and is defined at the surface ($z=0$). Between the surface and the first grid level where the scalars and horizontal velocity components are defined ($z=0.5\Updelta z$), a constant-flux layer is assumed based on Monin–Obukhov similarity theory (see Maronga et al. (2015) for full description; here we only report the boundary conditions for the neutral stratification case). The vertical profile of the horizontal velocity magnitude (${\overline{u}}_h = ({\overline{u}}^2+{\overline{v}}^2)^{1/2}$) is given in the surface layer by

22 $$\begin{aligned} \frac{\partial {\overline{u}}_h}{\partial z} = \frac{u_*}{\kappa z} \end{aligned}$$

**Table 4:** Boundary conditions of the flow and the passive scalar fields in LES. The Monin–Obukhov similarity theory is shown as MOST, and explained above in Appendix 2

	Inlet	Outlet	Lateral	Bottom	Top
Velocity field	Periodic	Periodic	Periodic	MOST	($\frac{\partial u}{\partial z} =0 = \frac{\partial v}{\partial z}, w=0$)
Scalar field	Dirichlet($c=0$)	Neumann($\frac{\partial c}{\partial x} = 0$)	Dirichlet ($c=0$)	Neumann($\frac{\partial c}{\partial z} = 0$)	Neumann($\frac{\partial c}{\partial z} = 0$)

where $\kappa =0.4$ is the von Kármán constant. In PALM, $u_*$ is calculated from ${\overline{u}}_h$ at $z=0.5\Updelta z$ by integrating Eq. 22 over *z* from the roughness length $z_0$ to $z=0.5\Updelta z$. The definition of $u_*$ is as follows

23 $$\begin{aligned} u_* = \left[ \left( {\overline{u'w'}}_0 \right) ^2+ \left( {\overline{v'w'}}_0 \right) ^2 \right] ^{1/4}, \end{aligned}$$

and therefore the surface momentum fluxes can be obtained by integrating the following equations over *z* from $z_0$ to $z=0.5\Updelta z$

24 $$\begin{aligned} \frac{\partial {\overline{u}}}{\partial z} = \frac{-{\overline{u'w'}}_0}{u_*\kappa z}, \,\,\,\, \,\,\, \frac{\partial {\overline{v}}}{\partial z} = \frac{-{\overline{v'w'}}_0}{u_*\kappa z}. \end{aligned}$$

The components of the ground stress tensor are used to calculate the vertical derivative of the velocity components at the first grid node. These derivatives are used in the calculation of the shear production term of the SGS TKE, Eq. 20. Furthermore, the ground stress components are used in the ground treatment of the diffusion term, last term in (18b). At the top, Neumann boundary conditions are applied ($\partial u/\partial z = \partial v /\partial z = 0$).

Neumann boundary conditions ($\partial e /\partial z = 0$) are also used for the SGS TKE. Vertical velocity is assumed to be zero ($w=0$) at the surface and top boundaries, which implies using Neumann boundary conditions for the pressure.

For the passive scalar, PALM allowed only periodic boundary conditions in along-wind (*x*) and crosswind (*y*) directions if periodic boundary conditions were applied to the flow field. However, this was not suitable for the purpose of this study. Therefore the boundary conditions for the passive scalar in along-wind (*x*) and crosswind (*y*) directions were modified and tested thoroughly. In the inlet plane (the (*y*, *z*) plane corresponding to the first grid point in along-wind direction), Dirichlet boundary condition is used. This means that the scalar quantity is set to zero ($c=0$), therefore no entrance of scalar is allowed from the inlet side. From the opposite side (outlet), the scalar can only exit. This is done by setting the scalar in ghost layers equal to that of the last grid point in (*x*), up to which the prognostic equation is solved ($\partial c/\partial x = 0$). This was done according to what PALM used for the scalar in conjunction with non periodic boundary conditions for velocity (outflow). Note that in PALM, some number of ghost layers are considered on each lateral boundary of the processor subdomains. The exchange of ghost layers is adapted to a dynamic number of ghost layers, depending on the applied advection scheme. In the crosswind (lateral) direction (*y*), the scalar is set to zero at both sides and also the ghost layers before the first grid point in *y* and after the last grid point in *y* (for which the prognostic equations are solved). We also used Neumann boundary condition on crosswind direction ($\partial c/\partial y = 0$), however the effect of the lateral boundaries on scalar dispersion statistics was found to be negligible for the range we explored here ($x/\delta \le 4$). At the bottom and top, the default PALM boundary conditions of the form of Neumann are used for scalar ($\partial c/\partial z = 0$). Boundary conditions of the velocity and scalar fields are detailed in Table 4.

## Appendix 3: The Meandering Plume Model

In the meandering plume model of Gifford (1959) the concentration p.d.f., *p*, can be written as

25 $$\begin{aligned} p(c;x,y,z) = \int _{0}^{\infty } \int _{-\infty }^{\infty } p_{cr}(c;x,y,z,y_{m},z_{m}) p_{m}(x,y_{m},z_{m}) dy_{m} dz_{m}, \end{aligned}$$

where *c* is the instantaneous concentration, $p_{cr}$ is the p.d.f. of the concentration in the meandering reference system centred in $(y_{m},z_{m})$ and $p_{m}$ is the p.d.f. of the position of the centre-of-mass. Assuming independence of meandering motions in crosswind and vertical directions and neglecting concentration fluctuations in the relative coordinate system, i.e. $p_{cr}=\delta _D{(c- \left\langle c_r \right\rangle })$, where $\delta _D$ here is the Dirac delta function and $\left\langle c_r \right\rangle$ is the mean concentration in relative coordinates (e.g., a Gaussian) which is a function of $\sigma _{ry}$ and $\sigma _{rz}$ (see Luhar et al. 2000; Cassiani and Giostra 2002; Marro et al. 2015), Eq. 25 can be rewritten as

26 $$\begin{aligned} p(c;x,y,z) = \int _{0}^{\infty } \int _{-\infty }^{\infty } \delta _D{(c- \left\langle c_r(x,y,z,y_m,z_m) \right\rangle )} p_{my}(x,y_m) p_{mz}(x,z_m) dy_m dz_m. \nonumber \\ \end{aligned}$$

$p_{m}=p_{my} p_{mz}$ and in the neutral baundary layer case these two p.d.f. can be taken as Gaussian functions of $\sigma _{my}$ and $\sigma _{mz}$. The *nth* concentration moment can be obtained (see Luhar et al. 2000; Marro et al. 2015) from

27 $$\begin{aligned} \begin{aligned} \langle c^n \rangle&= \left( \frac{Q}{2\pi \left\langle u_s \right\rangle \sigma _{ry}\sigma _{rz}} \right) ^n \frac{\sigma _{ry}}{{\left[ n \sigma _{my}^2 + \sigma _{ry}^2\right] }^{(1/2)}} \frac{\sigma _{rz}}{{\left[ n \sigma _{mz}^2 + \sigma _{rz}^2\right] }^{(1/2)}} \\&\quad \times \exp \left( - \frac{n (y-y_s)^2}{2( n \sigma _{my}^2 + \sigma _{ry}^2 )} \right) exp \left( - \frac{n (z-z_s)^2}{2( n \sigma _{mz}^2 + \sigma _{rz}^2 )} \right) , \end{aligned} \end{aligned}$$

where *Q* is the emitted mass at the source located in $y_s,z_s$. Using the relative dispersion and meandering generated by the LES, all the concentration moments can be calculated according to the meandering plume model. Equation 27 can be rewritten in the form of

28 $$\begin{aligned} \begin{aligned} \langle c^n \rangle&= \left( \frac{Q}{2\pi \left\langle u_s \right\rangle \sigma _{ry}\sigma _{rz}} \right) ^n \frac{1}{\left[ 1 + n M_y \right] ^{(1/2)}} \frac{1}{\left[ 1 + n M_z \right] ^{(1/2)}} \\&\quad \times \exp \left( - \frac{n (y-y_s)^2}{2 \sigma _{ry}^2 ( 1 + n M_y )} \right) \exp \left( - \frac{n (z-z_s)^2}{2\sigma _{rz}^2 ( 1 + n M_z)} \right) \end{aligned} \end{aligned}$$

with $M_y=\sigma _{my}^2 / \sigma _{ry}^2$ and $M_z=\sigma _{mz}^2 / \sigma _{rz}^2$. *Q* is $1~{\text {kg s}}^{-1}$, $u_s$ is the LES velocity at source elevation, $\sigma _r$ and $\sigma _m$ are relative dispersion and meandering, respectively as derived from the LES concentration data for crosswind and vertical directions, no parametrization is used. The contribution of internal concentration fluctuations, in the coordinate system relative to the local plume centre of mass, is neglected in this standard meandering formulation. In the simplified case with $M_y=M_z=M$ and in the plume centreline, Yee and Wilson (2000) demonstrated that

29 $$\begin{aligned} \sigma ^2_c = \left( \frac{(1+M)^2}{(1+2M)} -1 \right) \langle c \rangle ^2 . \end{aligned}$$

Therefore, with a constant absolute dispersion the variance of the concentration fluctuations will always increase with *M*.
